# Immunological Balance Is Associated with Clinical Outcome after Autologous Hematopoietic Stem Cell Transplantation in Type 1 Diabetes

**DOI:** 10.3389/fimmu.2017.00167

**Published:** 2017-02-22

**Authors:** Kelen C. R. Malmegrim, Júlia T. C. de Azevedo, Lucas C. M. Arruda, Joana R. F. Abreu, Carlos E. B. Couri, Gislane L. V. de Oliveira, Patricia V. B. Palma, Gabriela T. Scortegagna, Ana B. P. L. Stracieri, Daniela A. Moraes, Juliana B. E. Dias, Fabiano Pieroni, Renato Cunha, Luiza Guilherme, Nathália M. Santos, Milton C. Foss, Dimas T. Covas, Richard K. Burt, Belinda P. Simões, Júlio C. Voltarelli, Bart O. Roep, Maria C. Oliveira

**Affiliations:** ^1^Center for Cell-based Therapy, Regional Blood Center of Ribeirão Preto, Ribeirão Preto Medical School, University of São Paulo, Ribeirão Preto, Brazil; ^2^Department of Clinical, Toxicological and Bromatological Analysis, School of Pharmaceutical Sciences of Ribeirão Preto, University of São Paulo, Ribeirão Preto, Brazil; ^3^Department of Biochemistry and Immunology, Ribeirão Preto Medical School, University of São Paulo, Ribeirão Preto, Brazil; ^4^Department of Immunohematology and Blood Transfusion, Leiden University Medical Center, Leiden, Netherlands; ^5^Department of Internal Medicine, Ribeirão Preto Medical School, University of São Paulo, Ribeirão Preto, Brazil; ^6^Heart Institute (InCor), School of Medicine, University of São Paulo, São Paulo, Brazil; ^7^Division of Immunotherapy, Northwestern University Feinberg School of Medicine, Chicago, IL, USA; ^8^Department of Diabetes Immunology, Diabetes & Metabolism Research Institute at City of Hope, Duarte, CA, USA

**Keywords:** type 1 diabetes, autologous hematopoietic stem cell transplantation, immunoregulation, immune reconstitution, autoreactivity

## Abstract

Autologous hematopoietic stem cell transplantation (AHSCT) increases C-peptide levels and induces insulin independence in patients with type 1 diabetes. This study aimed to investigate how clinical outcomes may associate with the immunological status, especially concerning the balance between immunoregulation and autoreactivity. Twenty-one type 1 diabetes patients were monitored after AHSCT and assessed every 6 months for duration of insulin independence, C-peptide levels, frequencies of islet-specific autoreactive CD8^+^ T cells (CTL), regulatory lymphocyte subsets, thymic function, and T-cell repertoire diversity. In median follow-up of 78 (range 15–106) months, all patients became insulin-independent, resuming insulin after median of 43 (range 6–100) months. Patients were retrospectively divided into short- or prolonged-remission groups, according to duration of insulin independence. For the entire follow-up, CD3^+^CD4^+^ T-cell numbers remained lower than baseline in both groups, whereas CD3^+^CD8^+^ T-cell levels did not change, resulting in a CD4/CD8 ratio inversion. Memory CTL comprehended most of T cells detected on long-term follow-up of patients after AHSCT. B cells reconstituted to baseline levels at 2–3 months post-AHSCT in both patient groups. In the prolonged-remission-group, baseline islet-specific T-cell autoreactivity persisted after transplantation, but regulatory T cell counts increased. Patients with lower frequencies of autoreactive islet-specific T cells remained insulin-free longer and presented greater C-peptide levels than those with lower frequencies of these cells. Therefore, immune monitoring identified a subgroup of patients with superior clinical outcome of AHSCT. Our study shows that improved immunoregulation may balance autoreactivity endorsing better metabolic outcomes in patients with lower frequencies of islet-specific T cells. Development of new strategies of AHSCT is necessary to increase frequency and function of T and B regulatory cells and decrease efficiently autoreactive islet-specific T and B memory cells in type 1 diabetes patients undergoing transplantation.

## Introduction

Type 1 diabetes (T1D) results from T cell-mediated autoimmune destruction of β-cells from pancreatic islets, leading to insulin deficiency and hyperglycemia (1, 2). Beta cell destruction precedes overt diabetes in several months or years, and at diagnosis residual β-cells are still viable (3). In the past decades, several immunomodulatory clinical studies have attempted to modulate insulitis, aiming to preserve the remaining mass of β cells and the endogenous insulin production. Most studies showed little influence on β-cell function, at most keeping C-peptide levels stable while patients were under temporary immunosuppressive effects (4–16). In these studies, no discontinuation of exogenous insulin injections was reported during 1–2 years follow-up.

Autologous hematopoietic stem cell transplantation (AHSCT) has been investigated as a therapeutic approach for autoimmune diseases (17, 18). High dose immunosuppression, usually associated with T cell-specific depleting methods, is followed by infusion of previously cryopreserved autologous hematopoietic stem cells (HCS). In 2003, our group started a pioneer study on AHSCT for T1D. Recently, diagnosed patients underwent severe immunosuppression, aiming to preserve and perhaps improve β-cell function. Indeed, 21 out of 25 transplanted patients became insulin free after the procedure (19, 20).

The rationale involves “immune resetting,” which comprises non-specific abrogation of autoreactive T- and B-cell responses followed by successful reconstitution of a tolerant immune system (21–24). Key to the therapeutic success of AHSCT is to improve the immunoregulatory mechanisms, shifting the balance from autoimmunity to tolerance, therefore interrupting autoimmune destruction.

We herein describe updated clinical outcome and immune monitoring results of 21 type 1 diabetes patients treated with AHSCT, with median follow-up of 78 (range 15–106) months. Subjects presented different profiles of glycemic control after AHSCT. We were therefore able to identify two groups of patients, according to duration of insulin independence. In addition to C-peptide levels, patterns of immune reconstitution, thymic function, T-cell repertoire, and frequency of islet-specific autoreactive CD8^+^ T cells (CTL) were evaluated, aiming to detect immunological markers associated with different clinical outcomes after AHSCT.

## Materials and Methods

### Study Design and Patients

This clinical trial has been registered at http://clinicaltrials.gov (identifier: NCT00315133) and conducted in accordance with good clinical practice guidelines and approved by the institutional review board of the Ribeirão Preto Medical School of the University of São Paulo, Brazil (Comitê de Ética em Pesquisa do HCFMRP-USP, No 10095/2002). Written informed consent was received from each participant, in accordance with the Declaration of Helsinki, prior to inclusion in the study. A data safety monitoring board and an independent medical monitor provided additional study oversight.

**Figure 1:**
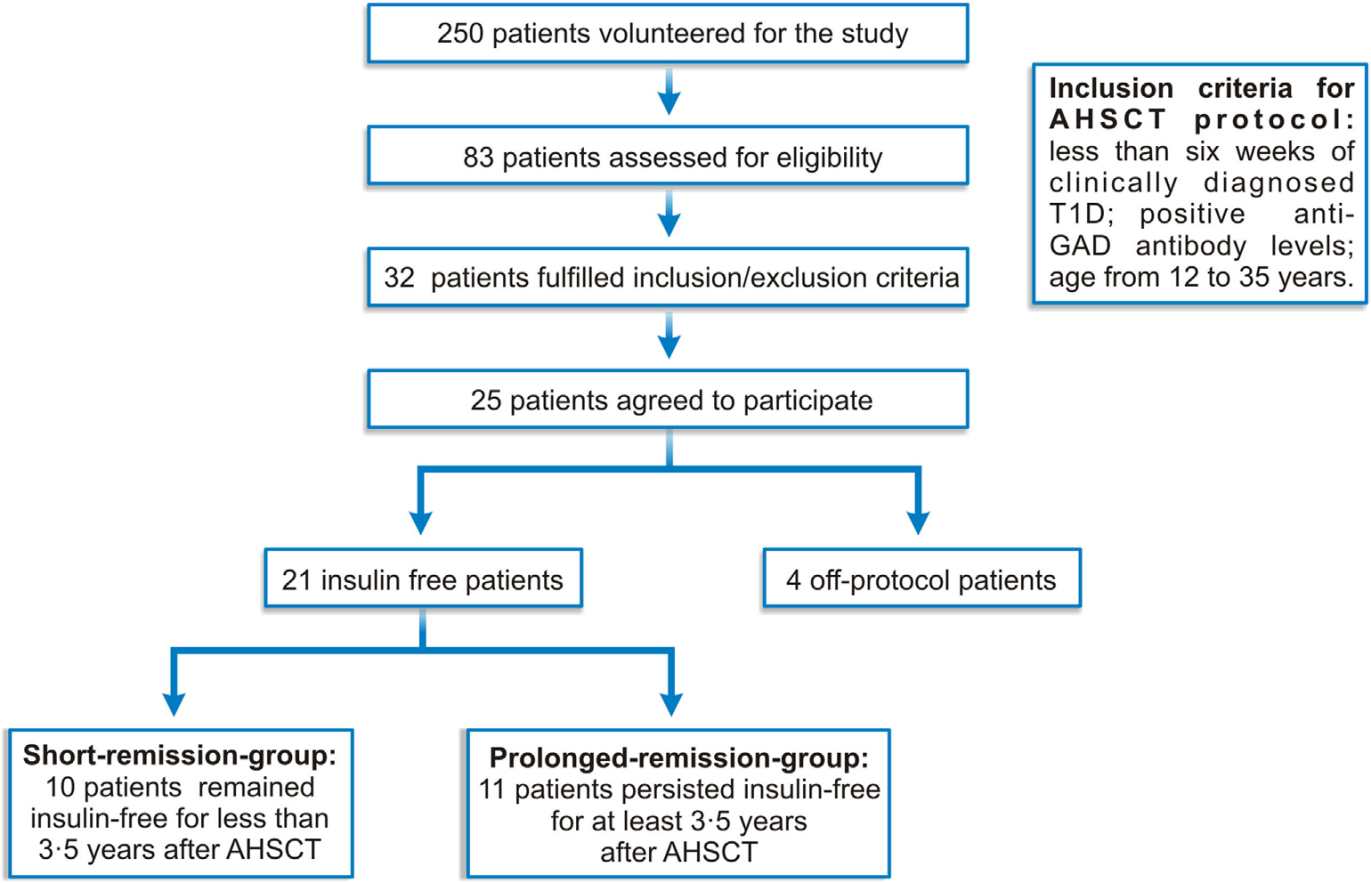
**Clinical protocol diagram**.

Twenty-five newly diagnosed type 1 diabetes patients fulfilled inclusion criteria and agreed to participate in this phase 2, single-arm, open-label AHSCT clinical trial and were prospectively followed-up for mean (SD) of 67.5 (26.9) months (19, 20) (Figure 1). Data from the first 23 patients have been previously described, as well as detailed inclusion and exclusion criteria (19, 20). Here, we report extended follow-up, including two additional patients.

Patients with less than 6 weeks of clinically diagnosed T1D and positive anti-glutamic acid decarboxylase (GAD) antibody levels had their HSC mobilized with 2 g/m^2^ cyclophosphamide and G-CSF and harvested through leukapheresis. Subsequently, patients were treated with 200 mg/kg cyclophosphamide plus 4.5 mg/kg rabbit anti-thymocyte globulin (rATG, Genzyme, USA), followed by infusion of unmanipulated, cryopreserved autologous HSC. Insulin doses were gradually reduced according to glycemic levels, aiming for glycated hemoglobin A_1c_ (HbA_1c_) of less than 7%, and fasting and 2 h after meal glucose levels of 120 and 140 mg/dL, respectively. Before and periodically after AHSCT, patients were evaluated, clinical data were registered, and blood samples were drawn for hematologic, metabolic, and immunological evaluations. Mean area under the curve (AUC) of C-peptide levels was measured during mixed-meal tolerance test, at baseline, 6 months and yearly after transplantation. From 25 patients, four were treated off-protocol and 21 were retrospectively analyzed for immunological parameters. The cohort of 21 patients was divided into upper and lower 50th percentile, based on duration of insulin freedom (Figure 1; Table 1).

**Table 1:** **Pretreatment and follow-up characteristics of type 1 diabetes patients undergoing non-myeloablative AHSCT**.

Patient no./sex	Age, years	Race	HLA class I	HLA class II	Blood glucose at diagnosis, mg/dL	Anti-GAD, at diagnosis, U/mL	A1C pretreatment, %	BMI at diagnosis, kg/m^2^	Insulin dose pre-mobilization, IU/kg/d	Insulin dose at last visit (IU/kg/d)	Follow-up, mo^a^	Time free from insulin, mo
**Short-remission-group (<3.5 years)^e^**												
7/F	20	White	A*02, *03/B*35, *44	DRB1*04, *12/DQB1*0302, *0301	391	4.0	10.0	16.8	0.44	0.36	91	7 (T)
10/F	17	White	A*03/B*14, *35	DRB1*01/DQB1*0501	612	44.0	8.9	20.1	0.29	0.26	87	9 (T)
11/M	16	Biracial^b^	A*01, *11/B*08, *35	DRB1*03, *04/DQB1*0201, *0302	130	11.0	5.4	17.8	0.13	0.34	15^c^	12 (T)
15/M	16	White	A*02, *03/B*18, *27	DRB1*01, *03/DQB1*0201, *0501	291	21.1	10.1	16.6	0.44	0.25	77	9 (T)
16/M	16	White	A*01, *02/B*08, *44	DRB1*03*04/DQB1*0201 *0302	384	5.3	8.4	18.3	0.56	0.30	66	29 (T)
18/M	21	White	A*02, *32/B*18, *44	DRB1*04/DQB1*0302	324	7.0	9.1	17.8	0.59	0.18	38^c^	23 (T)
19/M	15	White	A*26, *29/B*15, *40	DRB1*09/DQB1*0302	793	1.1	9.1	18.4	0.50	0.28	63	15 (T)
22/F	15	Asian American	A*02, *29/B*44, *54	DRB1*04, *12/DQB1*0302, *0302	390	10.0	11.6	22.6	0.33	0.80	57	9 (T)
23/M	22	White	A*02, *03/B*50, *51	DRB1*03/DQB1*0201	250	14	10.0	20.0	0.54	0.30	55	6 (T)
24/M	19	White	A*03, *25/B*44, *49	DRB1*03, *04/ND	280	1.5	9.9	21.5	0.12	0.38	45	7 (T)
Mean (SD)	17.70 (2.58)				384.5 (190)	11.90 (12.82)	9.25 (1.62)	18.99 (1.99)	0.39 (0.17)	0.35 (0.17)	59.4 (23.07)	^d^
**Prolonged-remission-group (≥3.5 years)^e^**												
2/M	27	Black	A*03, *30/B*18, *35	DRB1*03, *04/DQB1 *0201, *0302	589	49.0	7.5	22.9	0.34	0.16	106	47 (T)
3/M	21	Biracial^b^	A*03, *29/B*14, *14	DRB1*03, *04/DQB1 *0201, *0302	381	1.1	9.3	19.0	0.27	0	104	100 (C)
4/M	15	White	A*02/B*35, *58	DRB1*01, *07/DQB1 *0201, *0501	321	22.0	8.0	23.0	0.23	0.24	104	43 (T)
5/M	16	White	A*02, *23/B*07, *44	DRB1*04, *10/DQB1*0302, *0501	404	51.0	7.7	17.5	0.38	0	95	94 (C)
6/M	14	White	A*02, *29/B*07*44	DRB1*01, *03/DQB1*0201, *0501	504	17.0	7.3	23.4	0.42	0.44	71^c^	60 (T)
8/M	16	Biracial^b^	A*02/B*18, *44	DRB1*03, *04/DQB1*0201, *0302	314	48.0	5.4	17.6	0.55	0.41	88	44 (T)
9/F	18	White	A*02, *26/B*15, *58	DRB1*03, *13/DQB1*0201, *0602	330	102.0	6.7	19.1	0.35	0.40	88	61 (T)
12/F	14	Biracial^b^	A*01, *24/B*39, *44	DRB1*01, *04/DQB1*0302, *0501	581	11.0	8.1	19.8	0.45	0.33	80	66 (T)
13/M	24	White	A*24/B*18, *35	DRB1*03/DQB1*0201	269	24.0	8.1	18.4	0.58	0.11	79	67 (T)
14/M	31	White	A*03/B*41, *44	DRB1*04, *04/DQB1*0302, *0402	273	37.0	7.8	22.1	0.37	0.20	78	66 (T)
17/M	17	White	A*02, *68/B*35, *47	DRB1*04*08/DQB1*0302 *0402	236	12.0	9.0	20.7	0.21	0	65	64 (C)
Mean (SD)	19.36 (5.7)				382 (124·43)	34·01 (28·23)	7·72 (1·06)	20·32 (2·22)	0·38 (0·12)	0·21 (0·17)	87·09 (13·92)	^d^

*C, continuously; GAD, glutamic acid decarboxylase; T, transiently; ND, non-determined*.

*^a^Since mobilization regimen*.

*^b^Patients self-identified as having both black and white racial parentage*.

*^c^Patients lost follow-up*.

*^d^For the short-remission group, mean (SD) was 12.6 (7.66) months, and median (range) was 9 (6–29) months. For the prolonged-remission group, mean (SD) was 64.73 (18.34) months, and median (range) was 64 (43.100) months. For the cohort of 21 patients that experienced periods free from insulin post-autologous hematopoietic stem cell transplantation (AHSCT), mean (SD) was 39.9 (30.1) months, and the median (range) was 43 (6–100) months*.

^e^The cohort of 21 patients was divided into upper and lower 50th percentile, based on duration of insulin freedom. Patients that remained insulin free for less than 3.5 years after AHSCT were named “short-remission group,” and those that persisted insulin independent for at least 3.5 years were named “prolonged-remission group.”

*Patients were numbered according to transplantation day*.

### Immunophenotypic Analyses

Peripheral whole blood samples (150 µL) from 21 patients were immunophenotyped by flow cytometry, with previously titrated monoclonal antibodies (Becton-Dickinson, San Jose, CA, USA, EUA; Figures S1–S3 in Supplementary Material). Cells were analyzed with BD Biosciences FACSCalibur flow cytometer (Becton-Dickinson, San Jose, CA, USA, EUA). Gating strategies are shown (Table S1 in Supplementary Material). Thirty-thousand events per sample were acquired for each subset and 100,000 events for immunoregulatory T cells. Results were expressed as absolute cell numbers (cells per microliter).

### T-Cell Receptor Excision Circles (TRECs)

Peripheral blood mononuclear cells (PBMCs) from 18 patients were isolated by Ficoll-Hypaque™ density gradient centrifugation (Amersham-Pharmacia, Uppsala, Sweden). DNA was purified using DNeasy Blood & Tissue kit (Qiagen, Valencia, CA, USA) according to the manufacturer’s recommendations. DNA was diluted to concentration of 100 ng/μL and used for quantification of TREC molecules (25). Absolute quantification of TRECs (target gene) and albumin (endogenous gene control) in PBMCs was performed by real-time PCR, using TaqMan^®^ reagents and 7500 Real-Time PCR system (Applied Biosystems, Foster City, CA, USA). For each real-time PCR reaction, standard curves of TREC and endogenous genes were prepared. Plasmids containing TREC or albumin genes were kindly provided by Dr. Ngai Ka-Leung (Northwestern University, EUA). All experiments were performed in duplicates. The number of TREC molecules from each sample was divided by the number of endogenous gene copies (for normalization). Results were expressed as TREC molecules/100 ng DNA.

### Analysis of T-Cell Repertoire Diversity

Peripheral blood mononuclear cells from 21 patients were isolated by Ficoll-Hypaque™ density gradient centrifugation (Amersham-Pharmacia, Uppsala, Sweden). RNA was purified using Pure Link Mini Kit (Invitrogen Life Technologies, Carlsbad, CA, USA). Complementary DNA (cDNA) was synthesized from RNA using High Capacity cDNA Reverse Transcription Kit (Applied Biosystems, Foster City, CA, USA), according to the manufacturer’s recommendations. For further TCRBV CDR3 Length Spectratyping, TCR βV-βC segments were first PCR amplified with specific primers for the 24 variable TCR regions (24 βV families) and with one specific primer for the constant region of the TCR (βC primer), as described elsewhere (26, 27). The product from the first reaction underwent another PCR amplification (elongation or run-off reaction) that included an internal fluorescent βC primer. The segment containing the CDR3 region of the TCR was amplified (26, 27). Run-off reaction products were submitted to electrophorectic run in an automated DNA sequencer (ABI 3500xL Genetic Analyzer, Applied Biosystems, Foster City, CA, USA). Data were analyzed by Gene Mapper™ software (Applied Biosystems, Foster City, CA, USA), and distribution graphs of CDR3 segments were generated (26, 27). Graphs represent fluorescence intensity in arbitrary units according to the size of the CDR3 region in base pairs. Considering that the positions of the βV and βC primers are fixed, the size of PCR product labeled βV-βC depends on the size of the VDJ junction of each specific TCR. The size of CDR3 segment with 10 amino acid residues for each Vβ has been previously estimated (26, 27).

We then calculated the diversity of the TCR repertoire by the complexity score (CS) (28). Each Vβ family was classified from 0 to 8, based on the degree of TCR complexity/diversity. A score of 0 was given if the Vβ family was absent, of 1 if the Vβ family presented only one CDR3 peak, of 2 if the family presented two peaks, and subsequently. The score 8 was given to a spectratype with 8 or more peaks. The overall CS was calculated by the sum of the CSs of each Vβ family (28).

### Quantification of Islet-Specific CD8^+^ T Cells by Combinatorial Quantum Dot (Qdot) Approach

Multimeric peptide–MHC (pMHC) complexes were prepared as previously described (29). Briefly, recombinant HLA-A2 and human β2M were solubilized and injected together with each synthetic peptide into a refolding buffer. Multimeric pMHC complexes were produced by addition of streptavidin-conjugated Qdots (Invitrogen, Breda, The Netherlands) to achieve a 1:20 streptavidin-Qdot/biotinylated pMHC ratio. Qdot-585, -605, -655, -705, and -800 were used. PBMCs from HLA-A2 positive subjects were stained with a mixture containing six HLA-A2-associated diabetes epitopes (Table 2). Cells (2 × 10^6^) were stained simultaneously with all Qdot-labeled pMHC multimers (0.1 µg of each). Subsequently, 10 µL allophycocyanin-labeled anti-CD8 and 10 µL fluorescein isothiocyanate-labeled anti-CD4, -CD14, -CD16, -CD20, and -CD40 antibodies (Becton Dickinson, Franklin Lakes, NJ, USA) were added. Cells were resuspended in PBS/0.5% BSA containing 7-aminoactinomycin-D (7-AAD; eBioscience, San Diego, CA, USA) to exclude dead cells and analyzed using the LSRII (Becton Dickinson).

**Table 2:** **Combinations of quantum dot (Qdot) labeled HLA-A2 multimers**.

	Origin	Position/protein	Sequence	Signal
Cytomegalovirus	CMV	pp65	NLVPMVATV	Qdot 585 + 800
Epstein–Barr virus	EBV	LMP2	CLGGLLTMV	Qdot 585 + 800
Measles	measles	H250	SMYRVFEVGV	Qdot 585 + 800
HLA-A2	HLA-A2	140–149	YAYDGKDYIA	Qdot 585 + 605
Insulin	Insulin	B 10–18	HLVEALYLV	Qdot 605 + 655
Preproinsulin	PPI	15–24	ALWGPDPAAA	Qdot 705 + 655
Glutamic acid decarboxylase	GAD65	114–123	VMNILLQYVV	Qdot 800 + 655
Insulinoma-associated protein 2	IA-2	797–805	MVWESGCTV	Qdot 705 + 605
Islet-specificglucose-6-phosphatase catalytic subunit-related protein	IGRP	265–273	VLFGLGFAI	Qdot 800 + 605
Islet amyloid polypeptide	ppIAPP	5–13	KLQVFLIVL	Qdot 705 + 800

### Data Analysis

Patients were retrospectively divided into two groups, based on duration of insulin independence. The median was chosen as cut point because it dissected the patients into two groups of equal size who presented sustained vs short-lived response to therapy. Patients above the median duration of insulin independence were named “long-remission group” and those below, “short-remission group.” Further data analyses were performed in each of the subgroups, as well as in the undivided cohort of patients. For each patient, in the respective periods, C-peptide levels were calculated by mean AUC, which was estimated by the trapezium rule. The linear regression mixed model was used to analyze data from AUC, immune reconstitution, and frequency of autoreactive CTL (29). This method allowed for multiple longitudinal observations per individual across a baseline period, and subsequent time points after transplantation. The linear mixed models are composed of random and fixed effects. This model applies for analysis of data on which responses are grouped (more than one measure to the same individual), and the assumption of independence between observations in the same group is not adequate. The fixed effects are groups and periods. The random effects are associated with patients, since it is necessary to control correlations between repeated measures. For variable frequency, we used a logarithmic transformation to fit the data to the proposed model. Kaplan–Meier method was used to calculate insulin-free survival curve. Data analysis was performed using SAS^®^9.0 statistical software (SAS Institute Inc., Cary, NC, USA). Statistical significance was set at *p* < 0.05.

## Results

### AHSCT Induces Long-term Insulin Independence and Increases C-Peptide Levels

Twenty-five patients were enrolled between November 2003 and April 2010, and followed until 2014. Patient characteristics have been reported previously (19, 20) and are summarized (Table 1; Table S2 in Supplementary Material; Figure 1). Two patients had presented with ketoacidosis at diagnosis, and two others inadvertently received steroids during transplant procedure (Figure 1; Table S3 in Supplementary Material). These off-protocol patients were excluded from immunological analyses, allowing us to study a more homogeneous group of patients, with similar clinical and metabolic characteristics and also treated under the same transplant protocol. In fact, these four patients did not experience any period of insulin freedom after AHSCT. Details on this subpopulation of patients have been published (19, 20).

In the remaining patients, insulin doses were progressively reduced after AHSCT, based on glycemic levels, until complete suspension. Twenty-one patients became insulin free for median of 43 months (6–100 months) after AHSCT, and three of these patients are still continuously free from insulin (Table 1; Table S4 in Supplementary Material). Ten patients remained insulin free for less than 3.5 years after AHSCT and thus were named “short-remission group.” Eleven patients persisted insulin free for at least 3.5 years, therefore labeled “prolonged-remission group.” From 30 to 42 months of follow-up, there was no change in insulin use status, whether patients being were insulin free or insulin-dependent (Table S4 in Supplementary Material). This observation indicates the different profiles of each group, short-term and long-term responders, and reinforces the importance of investigating possible mechanistic differences between groups.

**Figure 2:**
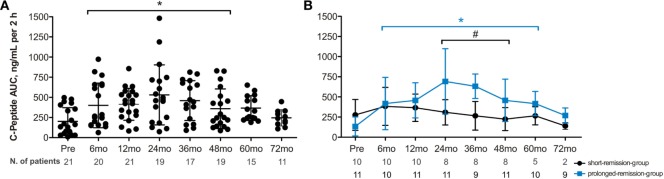
**Time course of total area under the curve of C-peptide levels during mixed-meal tolerance test in type 1 diabetes following autologous hematopoietic stem cell transplantation (AHSCT)**. **(A)** C-peptide levels in 21 type 1 diabetes patients who experienced any period free from insulin post-AHSCT. **(B)** C-peptide levels in 11 patients with prolonged remission and 10 patients with short remission after AHSCT. Statistical analysis was performed using a model of multiple regression of mixed effects. Data are shown as mean ± SD; mo, months. *p < 0.05: prolonged-remission group at cited period *vs* pretransplantation period. *p < 0.05: short-remission group at cited period *vs* pretransplantation period. ^#^*p* < 0.05 between the groups at cited period. Pre, pretransplantation period; mo, months. To convert C-peptide to nanomoles per liter, multiply by 0.331.

Mean (SD) AUC of C-peptide levels significantly increased from baseline compared with 6, 12, 24, 36, and 48 months post-AHSCT (Figures 2A,B; Table S5 in Supplementary Material). Furthermore, although at 60 and 72 months most patients had already resumed insulin, C-peptide levels were similar than those from before transplant. Moreover, when compared with baseline, the prolonged-remission group presented significant increase of C-peptide levels from 6 to 60 months after AHSCT, while the short-remission group showed stabilized C-peptide levels along same follow-up (Figure 2B; Figure S4 in Supplementary Material).

### Baseline Autoreactivity Predicts Clinical Outcomes after AHSCT

The profile of pancreas-infiltrating lymphocytes is partly mirrored in the peripheral blood of patients (29). Islet-autoreactive CTL have been detected in insulitic lesions from patients diagnosed with TID (30). These data indicate that measuring islet-specific CTL in peripheral blood may represent an excellent biomarker for treatment intervention. Thus, we first evaluated whether autoreactive islet-specific CD8^+^ T cell (aCTL) frequencies were associated with clinical outcomes of transplanted patients.

**Figure 3:**
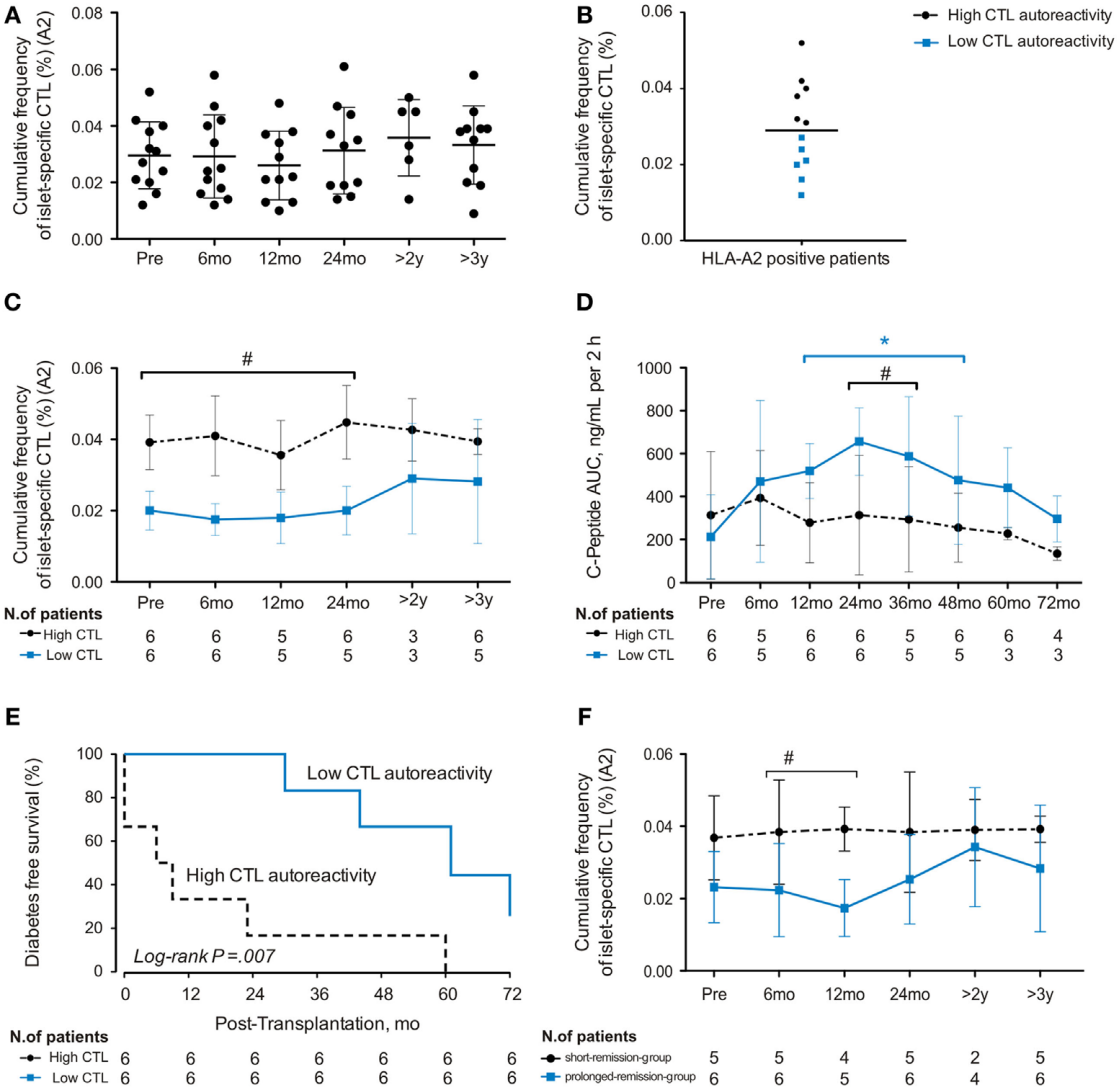
**Cumulative frequency of autoreactive islet-specific CD8^+^ T cells (CTL) at baseline predicts clinical outcome of autologous hematopoietic stem cell transplantation (AHSCT) in type 1 diabetes patients**. **(A)** Cumulative frequency (%) and dynamics of autoreactive islet-specific CTL in 12 HLA-A2 positive patients. **(B)** Patients were divided in two groups with high or low CTL autoreactivity. **(C)** Dynamics of autoreactive CD8^+^ T-cell frequencies after AHSCT in six patients with high CTL autoreactivity and six patients with low CTL autoreactivity. **(D)** Time course of total area under the curve of C-peptide levels during mixed-meal tolerance test in six patients with high CTL autoreactivity and six patients with low CTL autoreactivity. **(E)** Diabetes-free survival according to autoreactivity frequencies at baseline. **(F)** Dynamics of autoreactive CTL frequencies after AHSCT in six patients with prolonged remission and five patients with short remission. Statistical analysis was performed using a model of multiple regression of mixed effects. *p < 0.05: prolonged-remission group at cited period *vs* pretransplantation period. *p < 0.05: short-remission group at cited period *vs* pretransplantation period. ^#^*p* < 0.05 between the groups at cited period. Pre, pretransplantation period; mo, months; y, years.

Cumulative frequencies of islet-specific CTL recognizing six different islet epitopes were determined over time (Figure 3; Table S6 in Supplementary Material). We did not detect significant changes in aCTL frequencies following transplantation (Figure 3A). Subsequently, cumulative frequencies of aCTLs were divided into upper and lower 50th percentile, clustering patients according to the CD8^+^ T-cell autoreactivity at baseline (Figure 3B; Table S7 in Supplementary Material). Initial frequencies only minimally changed after AHSCT, indicating that the conditioning regimen was not able to eliminate aCTLs (Figure 3C). Patients with higher CTL autoreactivity before AHSCT maintained significantly elevated cumulative frequencies of aCTLs up to 24 months after transplantation. Moreover, patients with low CTL autoreactivity demonstrated higher C-peptide levels after AHSCT (Figure 3D; Table S8 in Supplementary Material).

Further analyses of insulin-free patients indicated that those with higher cumulative aCTL frequencies at baseline relapsed earlier post-AHSCT than patients with lower autoreactivity (*p* = 0.007; Figure 3E). Indeed, all patients with lower islet autoreactivity at baseline remained free from insulin for at least 24 months after AHSCT, while 83% of patients with higher baseline levels of islet autoreactivity had already relapsed at this time point (Figure 3E). In summary, our data show that baseline cumulative frequency of aCTLs predicts duration of insulin independency after AHSCT in T1D patients.

We next evaluated the dynamics of aCTL frequencies in the short- and prolonged-remission groups after AHSCT. Those remaining insulin free for longer periods had persistently lower frequencies of aCTLs that differed significantly at 6 and 12 months posttransplant (Figure 3F).

### Thymic Function Reactivation after AHSCT

**Figure 4:**
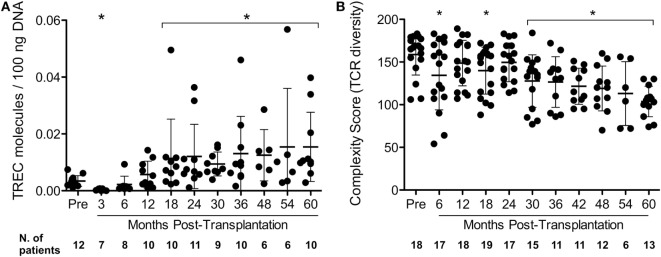
**Dynamics of thymic output and overall T-cell repertoire diversity in type 1 diabetes patients following autologous hematopoietic stem cell transplantation**. **(A)** T-cell receptor excision circle (TREC) levels over time. TREC levels were determined by real-time PCR. TREC levels are expressed as TREC molecules/100 ng DNA of peripheral blood mononuclear cells. **(B)** T-cell receptor repertoire diversity represented by complexity scores (CSs). T-cell receptor repertoire diversity was analyzed by TCRBV CDR3 Length Spectratyping, as detailed in Section “Materials and Methods.” The CS was determined by counting the number of CSs of each Vβ family. Data are shown as mean ± SD. *p < 0.05 between cited period *vs* pretransplantation period. Pre: pretransplantation.

Once AHSCT did not affect specific islet autoreactivity, we searched for other immunological mechanisms that might be involved in the control of β-cell destruction. Thymic production of naive T-cell populations (recent-thymic emigrants) was determined through analysis of signal-joint T-cell receptor excision circles. In our cohort of 21 patients, TREC levels were lower than baseline at 3 months, normalized at 12 months, and increased from 18 to 60 months after AHSCT (Figure 4A; Figure S5A and Table S9 in Supplementary Material). Concurrently, the T-cell repertoire diversity as defined by the CS was sustained until 24 months (Figure 4B; Figure S5B and Table S10 in Supplementary Material), progressively declining after 30 months post-AHSCT.

### Sustained CD4/CD8 Inversion after AHSCT

Lymphopenia was observed following transplantation in both groups, reflecting the immunosuppressive effect of the procedure (Figures S6A,B in Supplementary Material). We examined whether T- and B-cell subset reconstitution was associated with metabolic control of patients (Figure S6 in Supplementary Material). For the entire follow-up, CD3^+^CD4^+^ T-cell numbers remained lower than baseline in both groups (Figure S6C in Supplementary Material), whereas CD3^+^CD8^+^ T-cell levels did not change, resulting in a CD4/CD8 ratio inversion (Figures S6D,E in Supplementary Material). B cells reconstituted to baseline levels approximately 2–3 months post-AHSCT in both patient groups (Figure S6F in Supplementary Material).

**Figure 5:**
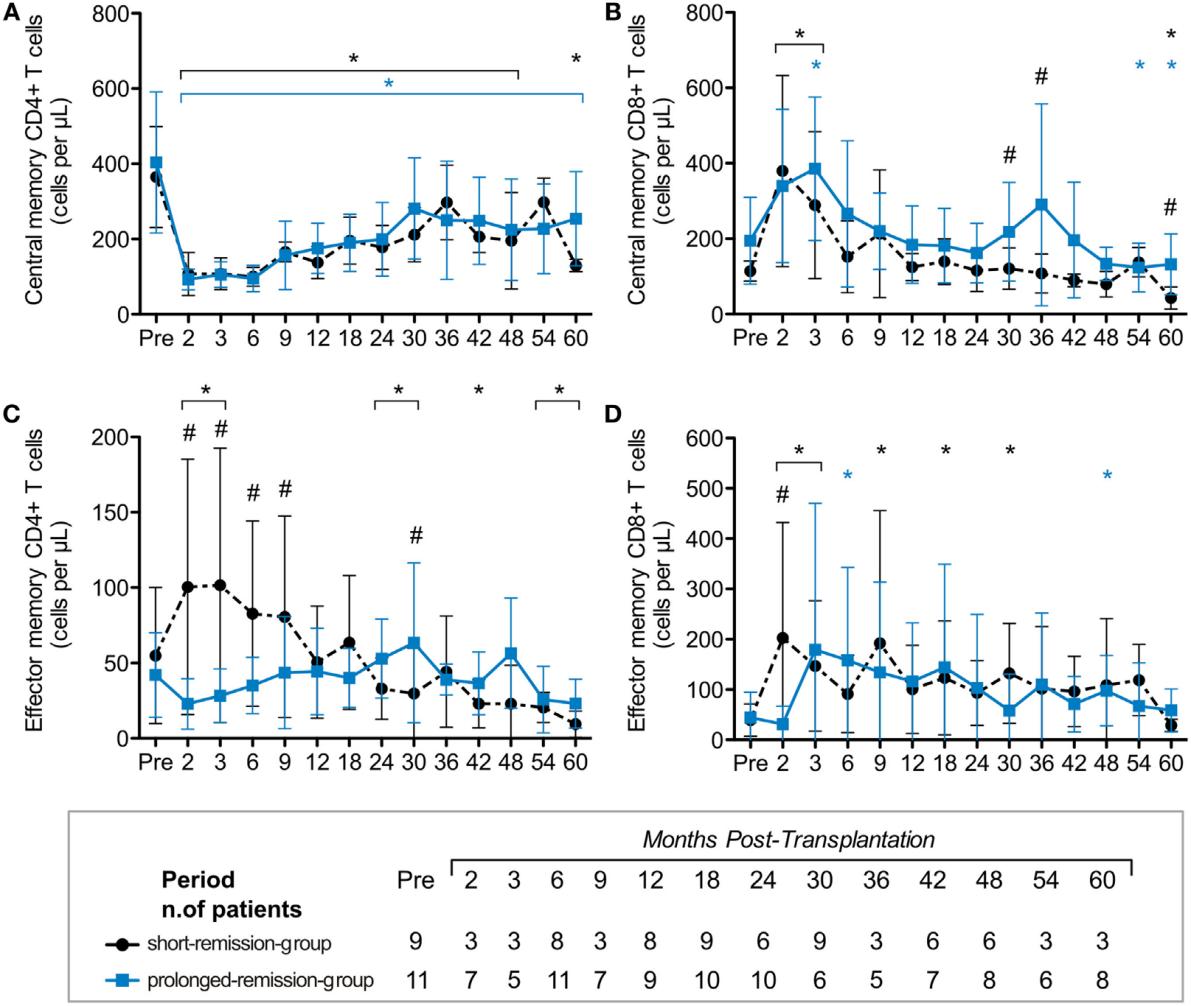
**Reconstitution kinetics of memory CD4^+^ and CD8^+^ T-cell subsets in type 1 diabetes patients following autologous hematopoietic stem cell transplantation (AHSCT)**. Reconstitution of absolute numbers (cells per microliter) of **(A)** central-memory CD4^+^CD27^+^CD45RO^+^ T cells, **(B)** central-memory CD8^+^CD27^+^CD45RO^+^ T cells, **(C)** effector memory CD4^+^CD27^−^CD45RO^+^ T cells, and **(D)** effector memory CD8^+^CD27^−^CD45RO^+^ T cells. Immunophenotyping of lymphocyte subsets was assessed by flow cytometry in samples of whole peripheral blood. Type 1 diabetes patients were divided in groups according to duration of insulin independence after treatment with AHSCT. Statistical analysis was performed using a model of multiple regression of mixed effects. *p < 0·05: prolonged-remission group at cited period *vs* pretransplantation period. *p < 0.05: short-remission group at cited period *vs* pretransplantation period. ^#^*p* < 0.05 between the groups at cited period. Pre, pretransplantation period.

We also investigated whether clinical response to AHSCT was associated with imbalanced distribution of memory T-cell subsets. In both patient groups, reconstitution to baseline numbers of central-memory CD4^+^ (CD4^+^T_CM_) cells was not detected throughout follow-up (Figure 5A), while overall central-memory CD8^+^ (CD8^+^T_CM_) cell counts increased at 2 and 3 months post-AHSCT, decreasing after 54 and 60 months (Figure 5B). The short-remission group had higher effector-memory CD4^+^ (CD4^+^T_EM_) cell counts at 2–9 months posttransplantation when compared with the prolonged-remission group (Figure 5C), while the prolonged-remission group presented higher CD8^+^T_CM_ values at 30, 36, and 60 months posttransplantation than the short-remission group. In both groups, effector-memory CD8^+^ (CD8^+^T_EM_) cell counts raised early after AHSCT (Figure 5D). In summary, memory CTL comprehended most of T cells detected on long-term follow-up of patients after AHSCT, indicating that the immunosuppressive regimen may not sufficiently target potentially autoreactive and pathogenic memory T cells.

### AHSCT Increases Regulatory T-Cell Counts

**Figure 6:**
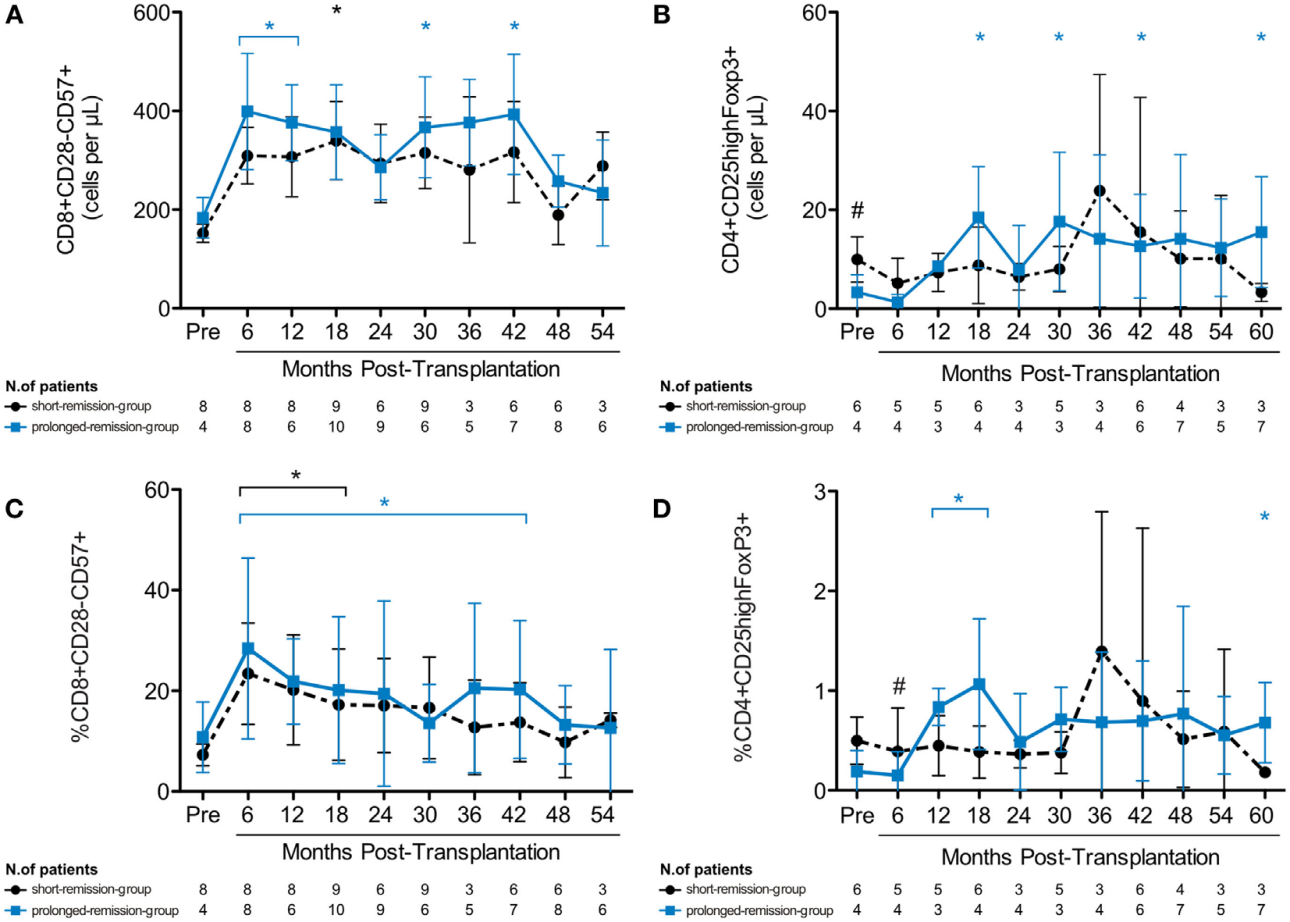
**Expansion of immunoregulatory T-cell subsets in type 1 diabetes patients after autologous hematopoietic stem cell transplantation (AHSCT)**. Reconstitution of absolute numbers of **(A)** CD8^+^CD28^−^CD57^+^ suppressor T cells and **(B)** regulatory CD4^+^CD25^high^FoxP3^+^ T cells. Frequency of **(C)** CD8^+^CD28^−^CD57^+^ suppressor T cells and **(D)** regulatory CD4^+^CD25^high^FoxP3^+^ T cells. The immunophenotypic analysis was assessed by flow cytometry of whole peripheral blood samples. Type 1 diabetes patients were divided in groups according to duration of insulin independence after treatment with AHSCT. Statistical analysis was performed using a model of multiple regression of mixed effects. *p < 0.05: prolonged-remission group at cited period *vs* pretransplantation period. *p < 0.05: short-remission group at cited period *vs* pretransplantation period. ^#^*p* < 0.05 between the groups at cited period. Pre, pretransplantation period.

Considering that total CD8 T cells were elevated after transplant, especially in those patients with longer clinical remission, we investigated the dynamics of CD8^+^CD28^−^CD57^+^ T cells post-AHSCT, due to their potential to decrease activity of autoreactive T cells (31). The prolonged-remission group showed increased numbers of CD8^+^CD28^−^CD57^+^ T cells at 6, 12, 30, and 42 months posttransplantation, compared with baseline levels, while the short-remission group showed elevated CD8^+^CD28^−^CD57^+^ cell counts only at 18 months post-AHSCT (Figure 6A). The percentage of CD8^+^CD28^−^CD57^+^ cells was increased from 6 to 42 months after transplantation (Figure 6C). Thus, during early years post-AHSCT, CD8^+^CD28^−^CD57^+^ and CD8^+^T_CM_ cell subsets constituted most of the T cells, especially in patients that presented better response to transplantation.

Next, we evaluated the reconstitution of CD4^+^CD25^hi^FoxP3^+^ regulatory T cells (Tregs) after AHSCT (32). At baseline, the short-remission group had higher absolute Treg numbers than the prolonged-remission group. However, after transplantation, the percentage and absolute numbers of Tregs significantly increased in the prolonged-remission group only (Figures 6B,D). Altogether, these data show that expansion of immunoregulatory T cells is associated with higher metabolic responsiveness to AHSCT, possibly by temporary reestablishment of self-tolerance.

## Discussion

Most patients in this study became insulin free for 3.5 years after transplantation and presented significant increments of C-peptide levels. This is different from the natural history of T1D and seems consonant with the hypothesis that a broad immunosuppressive approach could satisfactory control autoimmunity in diabetes (19, 20). In spite of not being a controlled trial, the longitudinal analysis and correlations among time free from insulin, frequency of islet-specific autoreactive CTLs and immunoregulatory mechanisms may guide future research protocols on the immunotherapies for T1D.

Our study demonstrates that although thymic reactivation was achieved, islet autoreactivity persisted after AHSCT. Nevertheless, most patients became insulin free and C-peptide levels remained higher than initial values for at least 4 years post-AHSCT, indicating temporary immunological balance and β-cell preservation. We contend that these results outweigh those of most immune intervention studies attempting to protect β cells in newly diagnosed T1D patients, while at best only delaying progressive loss of β-cell function, rather than improving endogenous insulin production (21).

Immunological mechanisms of AHSCT for autoimmune diseases have been described (22, 33–38). Here, we further contributed to the field by unraveling novel immune correlates associated with better metabolic responsiveness of type 1 diabetes patients to AHSCT: decreased effector-memory CD4^+^ T cells, expansion of immunoregulatory T cells, and lower frequencies of islet-specific autoreactive CTLs.

Shortly after AHSCT, patient’s immune reconstitution predominantly relies on peripheral homeostatic expansion of lymphocytes that survived the highly immunosuppressive regimen or that were reinfused with the stem cell graft (35, 36). A subpopulation of these cells may be effector-memory autoreactive cells and possibly associated with disease reactivation. Therefore, a fundamental step of AHSCT is proper elimination of the lymphoid compartment and consequent lymphopenia, which is essential to eradicate pathogenic autoreactive lymphocytes and thus abrogate autoimmune aggression to pancreatic β cells (21). However, in our study, we did not detect efficient elimination of memory T-cell subsets, including autoreactive CTLs. Yet, a subset of patients, mostly those in the lower 50th percentile of islet autoreactivity, presented better metabolic outcome, indicating that the detrimental effect of autoreactive CTL persistence might be counterbalanced by other immune mechanisms.

Thymic rebound, defined by volumetric enlargement and functional reactivation of the thymus following lymphoid depletion, has been previously reported after AHSCT (25, 34, 37). It may be measured by recovery of naive T-cell numbers and TREC levels (23, 37). Indeed, we show that TREC levels are decreased in both groups at 3 months, starting to increase again at 18 months after AHSCT, until the end of follow-up.

The diversity of the T-cell repertoire, however, presented a different pattern. Our results are in accordance with previous studies that demonstrated the reconstitution of a new and diverse T-cell repertoire in autoimmune patients up to 3 years after AHSCT (22, 34, 37). However, a late and gradual decline of the T-cell repertoire diversity occurred, indicating that a peripheral oligoclonal expansion of memory T cells, including autoreactive clones, may have surmounted the production of recent thymic emigrants.

Collectively, our results suggest that a diverse immune system was reconstituted in type 1 diabetes patients up to 30 months after AHSCT, contributing to temporary pancreatic β-cell preservation and insulin independence. However, after this time point, the overall diversity of the T-cell repertoire decreased and most of the long-lasting insulin-free patients resumed insulin, indicating that additional immune mechanisms might be operating and/or that the genetic background might have influenced the outcome. Despite the role of environmental factors as triggers for autoimmune diseases, the complexity of genetic predisposition might determine individual response to AHSCT and/or be responsible for late relapse of the disease (33).

In this setting, Tregs may have an important role in reestablishing self-tolerance and inducing long-term remissions after AHSCT (24, 32, 39, 40). Indeed, in short-term follow-up, infusions of Tregs decreased insulin requirements in six out of eight patients, two of which became insulin free (41). In our study, increased Tregs were only observed after AHSCT in the prolonged-remission group. It is known that Tregs modulate activated effector cells during early stages of antigen reexperiencing after transplantation, promoting self-tolerance (42). They may also inhibit spontaneous autoreactive proliferation in lymphopenic environments (43). In our study, patients’ insulin free for longer periods presented higher C-peptide levels, higher Treg numbers, and lower autoreactive CTL frequencies, suggesting that Tregs might have played a role in controlling autoimmunity. In the highly islet-reactive patients, however, immune regulatory mechanisms might have been insufficient to restrain β-cell destruction. Our data suggest that memory autoreactive CTLs were not satisfactorily depleted by the conditioning regimen and probably underwent peripheral expansion. This interpretation is supported by similar findings from a clinical trial assessing ATG therapy in new-onset T1D patients (13). Drugs more effectively targeting memory T cells may improve remission rates and preserve β-cell function. In fact, an elegant study showed that anti-CD2 antibody depletes central- and effector-memory cells, contributing to slower rate of β-cell destruction (14).

In accordance with the literature (34, 39), we observed expansion of CD8^+^CD28^−^CD57^+^ cells after AHSCT, a CD8^+^ subpopulation with immunosuppressive and immunoregulatory properties. Our work is the first to demonstrate that higher responsiveness to AHSCT and consequent insulin independence in T1D patients may be related to *in vivo* expansion of immunoregulatory cells. We recognize that functional assays with immunoregulatory cell subsets would be important to verify their suppressive capacity also *in vitro*. These investigations are planned for future studies.

Importantly, we were able to identify an immune correlate of treatment efficacy, as patients with low frequencies of autoreactive CTLs before transplant remained independent of insulin injections longer than patients with high frequencies these cells. Type 1 diabetes represents a heterogeneous disease in terms of low and high autoreactive T-cell frequencies, and therapeutic efficacy differs between patient subsets. Indeed, in the setting of islet transplantation, the rate of baseline cellular islet autoimmunity predicts clinical outcomes (29, 44). These data show that measurement of autoreactive CTL frequency in the peripheral blood may be useful to predict which group of patients will benefit most from the current transplant conditioning scheme and which may require more intense strategies.

Our study demonstrates encouraging metabolic outcomes in recent-diagnosed type 1 patients. Similar results were achieved by other independent centers, as well as by a preclinical study (45–50). It is known that C-peptide levels inversely correlate with the incidence of diabetic nephropathy, neuropathy, and hypoglycemia (44). We show that mean C-peptide levels remained higher than baseline long after transplant, indicating persistent effects of AHSCT on disease pathogenesis and possibly on the long-term outcome of these patients.

The main target of AHSCT for autoimmune diseases is to achieve immunological tolerance, which results from balance between autoreactivity and immunoregulatory mechanisms, in the context of a newly regenerated immune system. Nevertheless, new strategies are desired to boost T and B regulatory cell mechanisms and/or more efficiently eliminate autoreactive T and B memory cells in the AHSCT setting (14). To address this issue, new protocols of AHSCT for newly diagnosed T1D should be developed, including a three-drug immunosuppressive regimen (cyclophosphamide, fludarabine, and rATG), aiming to hit the memory T and B cell compartment harder and improve treatment outcomes. In addition, evaluation of the B-cell subsets and their function in the AHSCT setting for type 1 diabetes and other autoimmune diseases is warranted and may support future therapeutic approaches.

## Author Contributions

MO and KM are the guarantor of this work, i.e., had full access to all the data in the study and takes responsibility for the integrity of the data and the accuracy of the data analysis. Study concept and design: KM, CC, MO, BS, and RB. Acquisition of data: KM, CC, LA, JA, GO, PP, MO, AS, DM, FP, JD, RC, BS, MF, DC, JA, and BR. Analysis and interpretation of data: KM, CC, MO, LA, JA, BS, LG, RB, JA, and BR. Drafting of the manuscript: KM, LA, CC, BR, and MO. Critical revision of the manuscript for important intellectual content: KM, CC, MO, JA, LA, GO, PP, DC, LG, AS, DM, FP, RC, JD, MF, BS, RB, JA, and BR. Statistical analysis: KM, LA, and CC. Obtained funding: MO, KM, and CC. Administrative, technical, or material support: DC and BS. Study supervision: MO, KM, CC, BS, and BR.

## Conflict of Interest Statement

The authors declare that the research was conducted in the absence of any commercial or financial relationships that could be construed as a potential conflict of interest.
