# Supplementary material for: Immunological Balance Is Associated with Clinical Outcome after Autologous Hematopoietic Stem Cell Transplantation in Type 1 Diabetes
Source: Front Immunol. 2017 Feb 22;8:167. doi: 10.3389/fimmu.2017.00167 (PMC5319960; doi:10.3389/fimmu.2017.00167)
Supplement: Supplementary file 1 [file Data_Sheet_1.PDF]

SUPPLEMENTARY MATERIAL

Immunological balance is associated with clinical outcomes after autologous hematopoietic stem cell transplantation in type 1 diabetes patients

Kelen C. R. Malmegrim, Júlia T. C. de Azevedo, Lucas C. M. Arruda, Joana R. F. Abreu, Carlos E. B. Couri, Gislane L. V. de Oliveira, Patrícia V. B. Palma, Gabriela T. Scortegagna, Ana B. P. L. Stracieri, Daniela A. Moraes, Juliana B. E. Dias, Fabiano Pieroni, Renato Cunha, Nathália M. Santos, Luiza Guilherme, Milton C. Foss, Dimas T. Covas, Richard K. Burt, Belinda P. Simões, Júlio C. Voltarelli, Bart O. Roep, Maria C. Oliveira

*** Correspondence:** kelenfarias@fcfrp.usp.br

**Supplementary Figures**..		2
**Figure S1**	Immunophenotypic analysis of T and B subpopulations…………………………	2
**Figure S2**	Immunophenotypic analysis of memory T cell subpopulations…………………...	3
**Figure S3**	Immunophenotypic analysis of regulatory T cells………………………………	4
**Figure S4**	Time course of total area under the curve (AUC) of C-peptide levels during mixed-meal tolerance test in type 1 diabetes following AHSCT………………….	5
**Figure S5**	Dynamics of thymic output and overall T cell repertoire diversity in T1D following AHSCT..	6
**Figure S6**	Reconstitution kinetics of T and B cell subsets in T1D patients following AHSCT..	7
**Supplementary Tables**..		8
**Table S1.**	Gating strategy of T-cell subsets by flow cytometry...	8
**Table S2.**	Adverse effects of patients with T1D patients undergoing non-myeloablative AHSCT..	9
**Table S3.**	Pre-treatment and follow-up characteristics of off-protocol T1D patients…………..	10
**Table S4.**	Insulin-use status of T1D patients following non-myeloablative AHSCT.................	11
**Table S5.**	C-peptide levels in T1D patients submitted to AHSCT..	12
**Table S6.**	Cumulative frequency of islet-specific autoreactive CD8+ T-cells in T1D patients following AHSCT..	13
**Table S7.**	Baseline cumulative frequency of islet-specific autoreactive CD8+ T-cells in T1D patients..	14
**Table S8.**	C-peptide levels in T1D patients with High or Low CLT autoreactivity at baseline..	15
**Table S9.**	T-cell receptor excision circles levels in peripheral blood mononuclear cells of T1D patients following AHSCT...	16
**Table S10.**	T cell repertoire diversity in T1D patients following AHSCT..................................	17

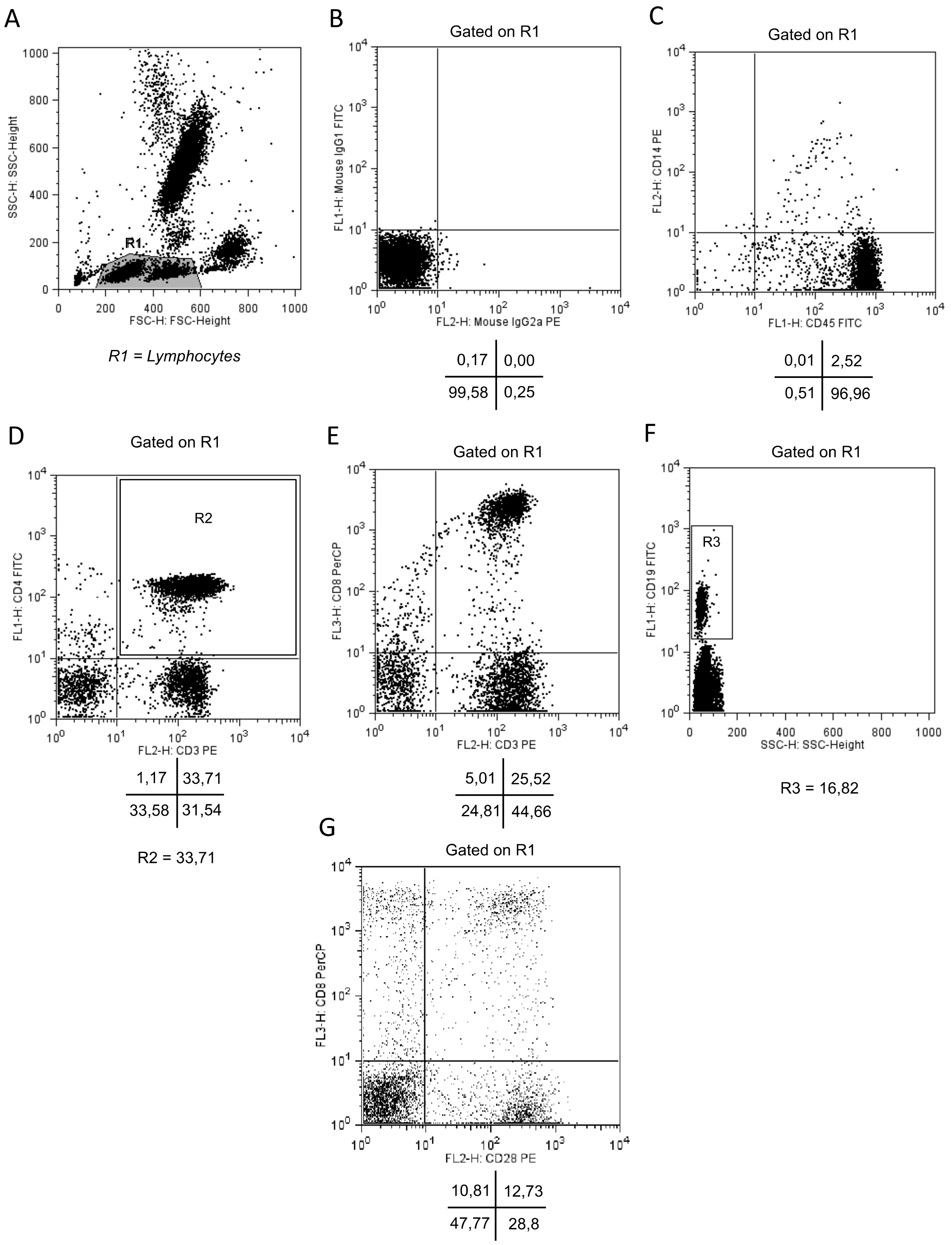

Supplementary Figure 1. Immunophenotypic analyses of T and B subpopulations. (A) During cell acquisition a gate (R1) was drawn in the lymphocyte population, based on the parameters of size (FSC) and granularity (SSC). (B) Percentage (%) of isotype control staining. Dot plots of FL1 versus FL2 were done. Subsequently, a R2 gate was drawn in the double-positive population for analysis of FL3 versus FL4. (C) Control gate to exclude monocytes from the analysis. Percent (%) of helper $CD3^{+}CD4^{+}$ T cells (D) cytotoxic T $CD3^{+}CD8^{+}$ T cells (E) $CD19^{+}$ B cells (F) $CD8^{+}CD28^{-}$ T cells (G).

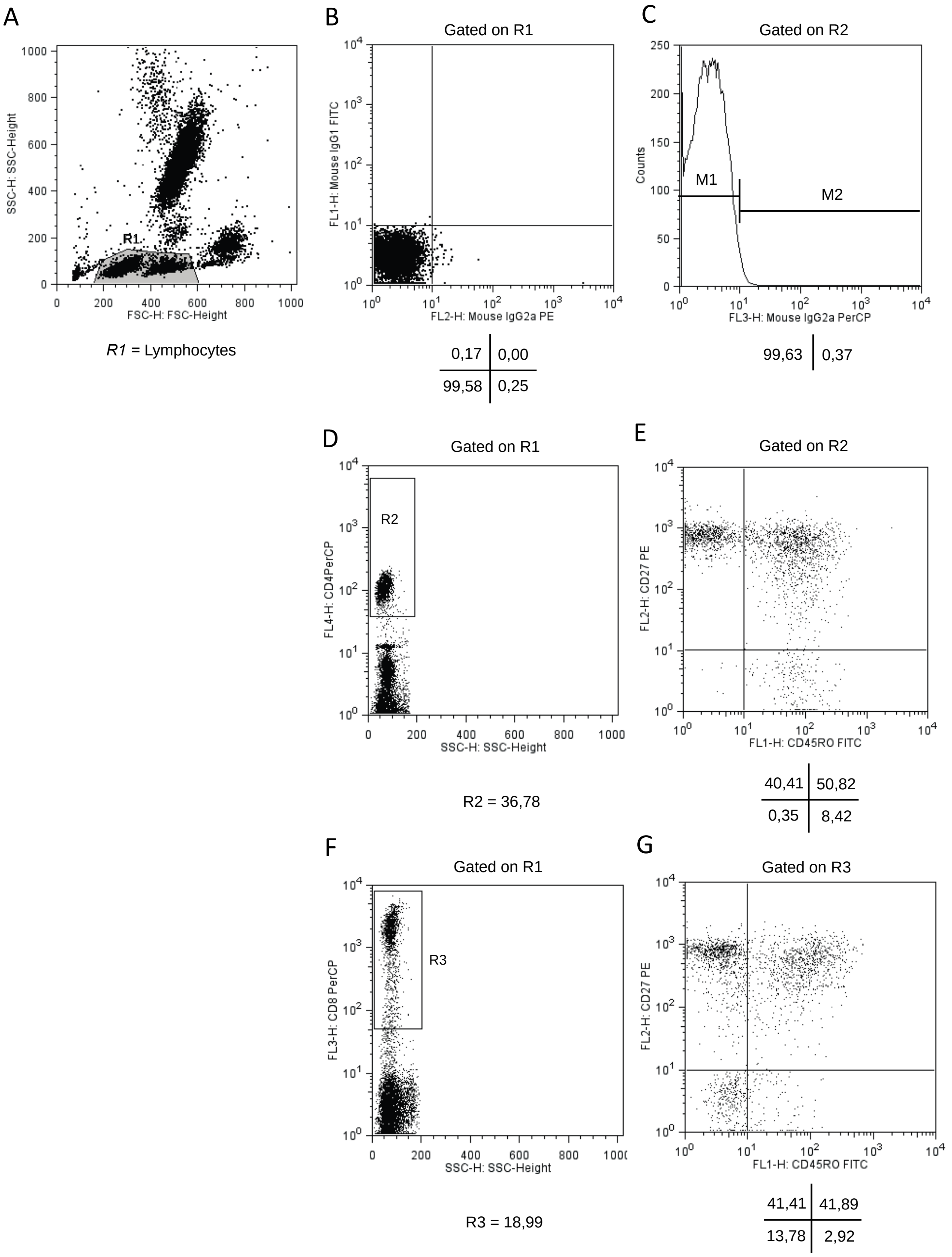

Supplementary Figure 2. Immunophenotypic analyses of memory T-cell subpopulations. (A) During cell acquisition a gate (R1) was drawn in the lymphocyte population, based on the parameters of size (FSC) and granularity (SSC). (B) Percentage (%) of isotype control staining. (C) Percentage (%) of isotype control staining of gate (R2). The $CD4^{+}$ (D) or $CD8^{+}$ (F) T cell subpopulations were selected by the gates R2 (FL4) ou R3 (FL3). Dot plots of FL1 versus FL2 were done to analyze the events of gate R2. Percentage of central memory $CD4^{+}CD27^{+}CD45RO^{+}$ T cells (upper right quadrant) and effector memory $CD4^{+}CD27^{-}CD45RO^{+}$ T cells (lower right quadrant) (E), central memory CD8+CD27+$CD45RO^{+}$ T cells (upper right quadrant) and effector memory $CD8^{+}CD27^{-}CD45RO^{+}$ T cells (lower right quadrant) (G).

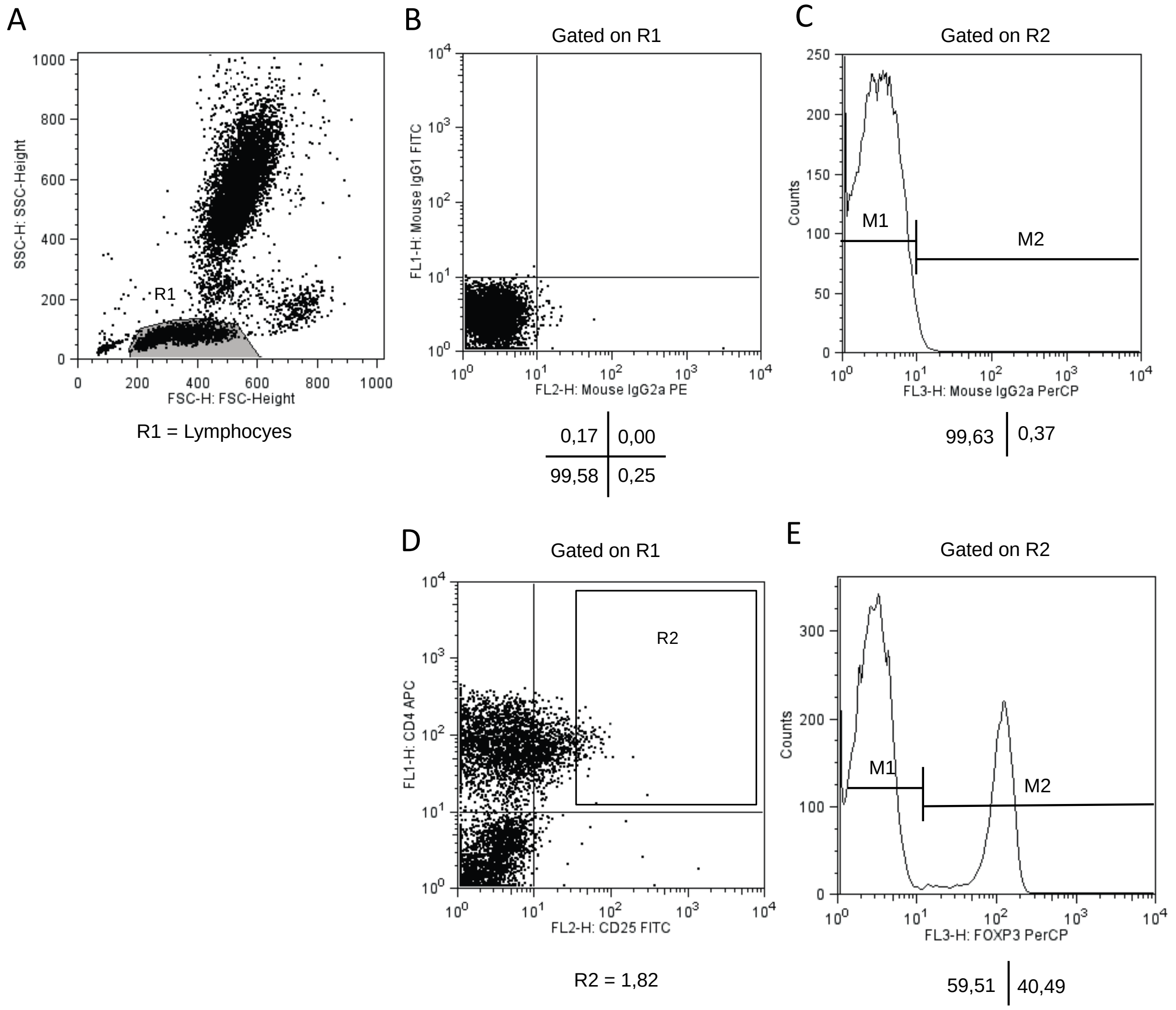

Supplementary Figure 3. Immunophenotypic analyses of regulatory T cells. (A) During cell acquisition a gate (R1) was drawn in the lymphocyte population, based on the parameters of size (FSC) and granularity (SSC). (B) Percentage (%) of isotype control staining. (C) Percentage (%) of isotype control staining of gate (R2). Dot plots of FL1 versus FL2 were done. Then, a R2 gate was drawn in the double-positive $CD4^{+}CD25^{high}$ T cells (D). Percentage (%) of regulatory $CD4^{+}CD25^{high}FOXP3^{+}$ T cells (E).

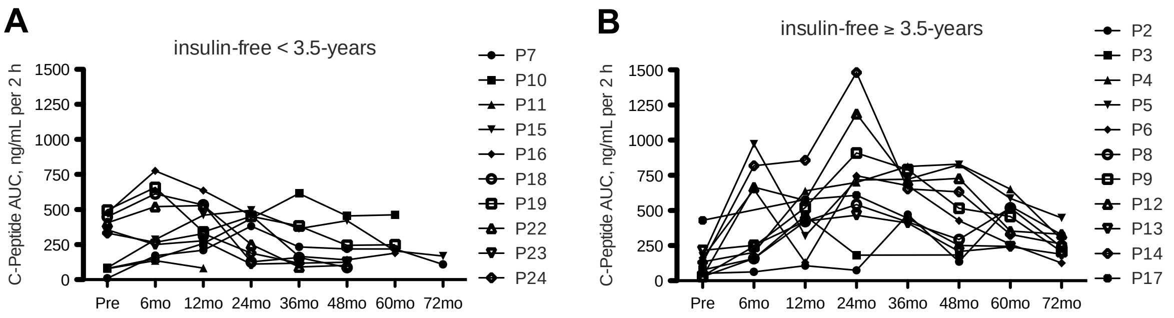

Supplementary Figure 4. Time course of total area under the curve (AUC) of C-peptide levels during mixed-meal tolerance test in type 1 diabetes following AHSCT. (A) Individual curves for C-peptide levels in the short- and (B) prolonged-remission-groups. Pre, pre-transplantation period; mo, months. To convert C-peptide to nmol/L, multiply by 0.331.

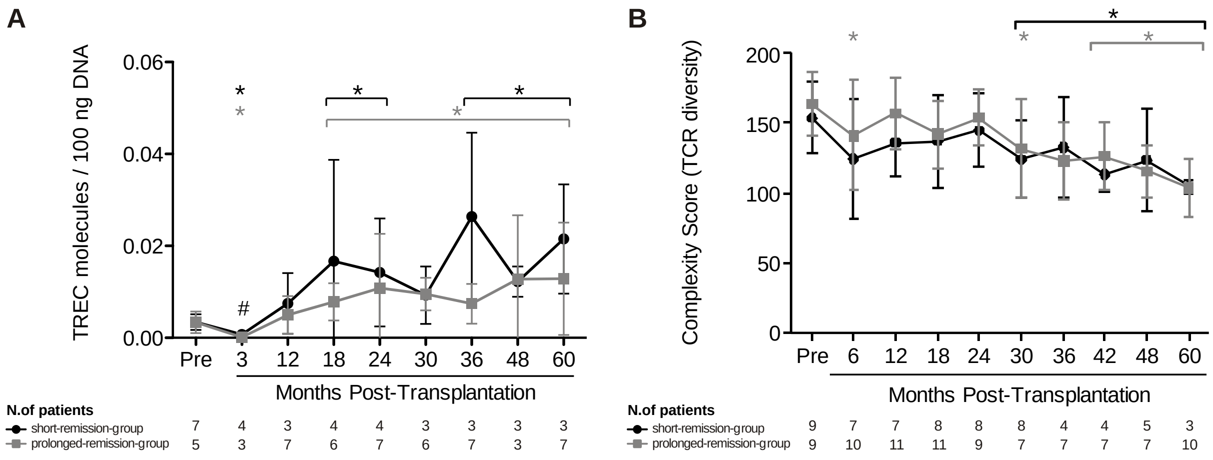

Supplementary Figure 5. Dynamics of thymic output and overall T-cell repertoire diversity in T1D patients following AHSCT. Peripheral blood mononuclear cells (PBMCs) from 11 insulin-free ≥3.5-years and 10 insulin-free <3.5-years groups of T1D patients were isolated by density gradient centrifugation. DNA was purified from PBMCs and the absolute quantification of T-cell receptor excision circles (TREC) levels (**A**) was performed by real-time PCR. (**B**) RNA was purified from PBMCs, cDNA was synthesized and analyzed by the TCRBV CDR3 Length Spectratyping method. T cell receptor repertoire diversity was assessed by the complexity score. Statistical analysis was performed using a model of multiple regression of mixed effects. ■, patients insulin-free for at least 3.5 years (≥3.5-years-group). ●, patients insulin-free less than 3.5 years (<3.5-years-group). *, *p*<0.05: ≥3.5-years-group at cited period vs pre-transplantation period. *, p<0.05: <3.5-years-group at cited period vs pre-transplantation period. #, *p*<0.05 between the groups at cited period. Pre, pre-transplantation period.

frontiers

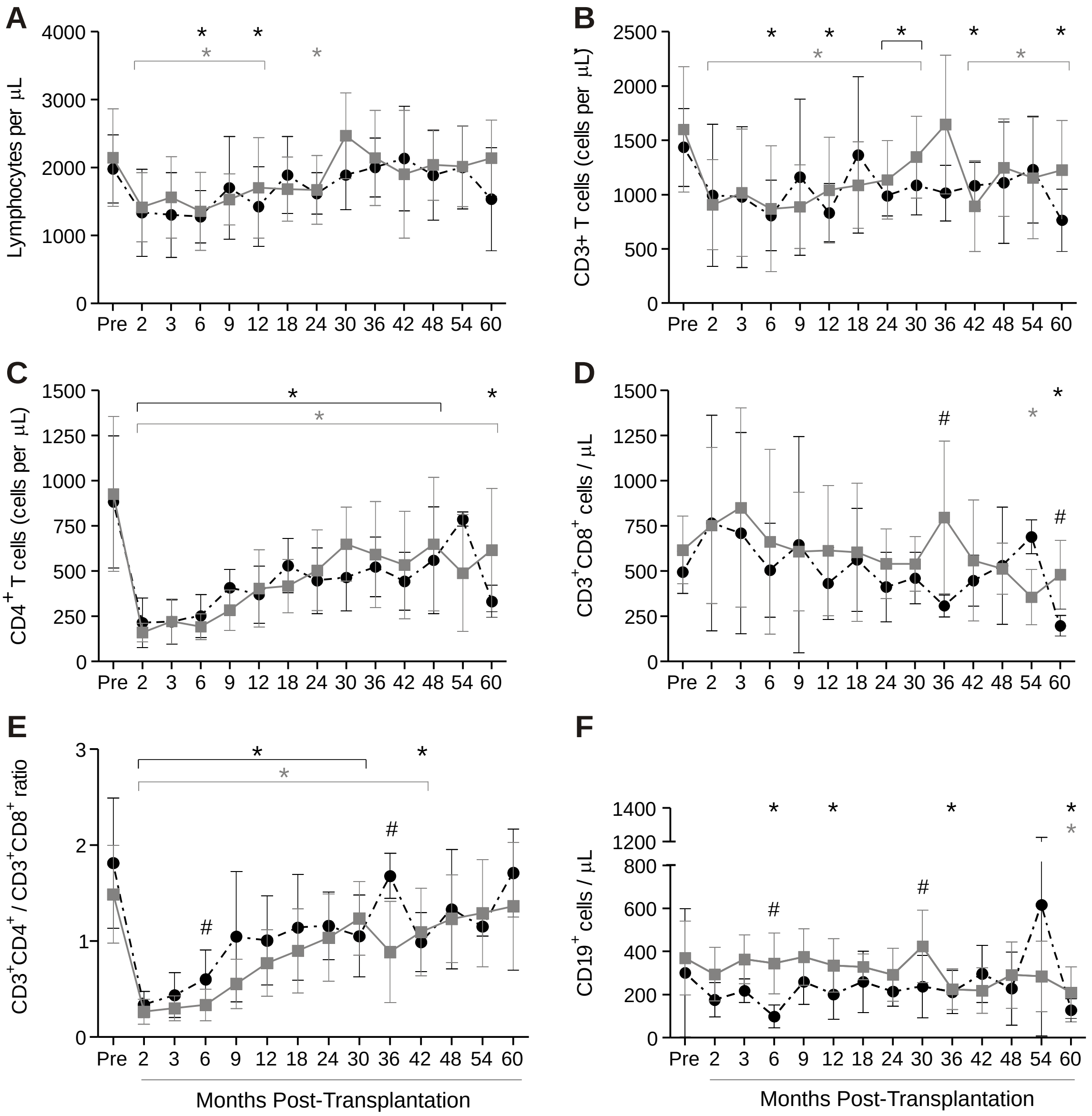

Period (months)	Pre	2	3	6	9	12	18	24	30	36	42	48	54	60
**N. of patients**														
short-remission-group	9	3	3	8	3	8	9	6	9	3	6	6	3	3
prolonged-remission-group	11	7	5	11	7	9	10	10	6	5	7	8	6	8

Supplementary Figure 6. Reconstitution kinetics of T and B cell subsets in type 1 diabetes patients following AHSCT. Reconstitution of absolute numbers (cells / µL) of total lymphocytes (**A**), CD3+ T-cells (**B**), CD3+CD4+ T-cells (**C**), CD3+CD8+ T-cells (**D**), CD4/CD8 ratio (**E**) and CD19+ B-cells (**F**). The immmunophenotypic analysis was assessed by flow cytometry of whole peripheral blood of 11 insulin-free ≥3.5-years and 10 insulin-free <3.5-years groups of T1D patients. T1D patients were divided in groups according to duration of insulin dependence after treatment with AHSCT. Statistical analysis was performed using a model of multiple regression of mixed effects. ■, patients insulin-free for at least 3.5 years (≥3.5-years-group). ●, patients insulin-free less than 3.5 years (<3.5-years-group). *, $p<0.05$: ≥3.5-years-group at cited period vs pre-transplantation period. *, p<0.05: <3.5-years-group at cited period vs pre-transplantation period. #, $p<0.05$ between the groups at cited period. Pre, pre-transplantation period.

Supplementary Table 1. Gating strategy of T-cell subsets by flow cytometry.

Lymphocyte subsets	FITC (Fluorescein iso-thiocyanate)	PE (Phycoerythrin)	PerCP (Peridinin-chlorophyll-protein complex)	APC (Allophycocyanin)
	**CD3**	**CD4**	**CD8**	**CD19**
CD3+ T cells	wbc→lymph→CD3+			
CD4+ T cells	wbc→lymph→CD3+CD4+			
CD8+ T cells	wbc→lymph→CD3+CD8+			
CD19+ B cells	wbc→lymph→CD19+			
**Naive and memory T cell subsets**	**CD45RO**	**CD27**	**CD8**	**CD4**
CD45ROnegCD27+ Naïve T cells	wbc→lymph→CD4+(or CD8+)→CD45ROnegCD27+			
CD45RO+CD27+ Central Memory T cells	wbc→lymph→CD4+(or CD8+)→CD45RO+CD27+			
CD45RO+CD27neg Effector Memory T cells	wbc→lymph→CD4+(or CD8+)→CD45RO+CD27neg			
**Regulatory CD4 T cells**	**CD25**	**FoxP3**		**CD4**
CD4+CD25highFoxP3+	wbc→lymph→CD4+CD25high→FoxP3+ cells			
**Regulatory CD8 T cells**	**CD28**	**CD57**	**CD8**	
CD8+CD28negCD57+	wbc→lymph→CD8+CD28neg→CD57+ cells			

frontiers

Supplementary Table 2. Adverse effects of patients with T1D patients undergoing non-myeloablative AHSCT.

Patient Number	Major acute adverse effects	Late adverse effects
1	None	None
2	Bilateral pneumonia	Graves' disease (2.8y)
3	None	Hypothyroidism + rhabdomyolysis (1y)
4	None	Transient leucopenia, Transient oligospermia
5	None	Transient oligospermia
6	None	Graves' disease (3.5y)
7	None	None
8	None	Graves' disease (4.5y), Transient oligospermia
9	None	None
10	None	Transient hypogonadism (1y),Mild SLE (4y)
11	None	None
12	None	None
13	None	Transient oligospermia
14	None	Transient oligospermia
15	None	Transient oligospermia
16	None	Transient oligospermia
17	None	Oligospermia
18	None	Oligospermia
19	None	Transient oligospermia
20	None	Oligospermia
21	None	None
22	Bilateral pneumonia	None
23	None	None
24	None	None
25	None	Graves' disease (1.8y)

SLE, Systemic Lupus Erythematosus; T1D, type 1 diabetes; AHSCT, autologous hematopoietic stem cell transplantation. Y: year.

frontiers

Supplementary Table 3. Pre-treatment and follow-up characteristics of the off-protocolT1D patients

Patient No./Sex	Age, y	Race	HLA class I	HLA class II	Blood glucose at diagnosis, mg/dl	Anti-GAD, at diagnosis, U/ml	A1C pre-treatment, %	BMI at diagnosis, Kg/m[2]	Insulin Dose Pre-mobilization, IU/kg/d	Insulin dose at last visit (IU/kg/d)	Follow-up, mo[a]	Time free from insulin, mo
1/M[b,c]	24	Biracial[d]	A*11,*24/ B*18, *35	DRB1*03, *04/ DQB1 *0201, *0302	477	36.0	7.6	22.6	0.51	1.7	12[e]	NS
20/M[c]	13	White	A*01, *02/ B*39, *55	DRB1*03, *04/ DQB1*0201, *0302	485	29.0	7.6	18.3	0.49	0.62	61	NS
21/F[b]	16	White	A*01, *24/ B*08, *51	DRB1*01, *04/ DQB1*0302, *0501	439	16.0	9.5	21.1	0.49	0.53	36[e]	NS
25/F[b]	16	White	A*02, *25/ B*15, *49	DRB1*04, *04 /ND	337	14.8	9.1	22.6	0.41	0.53	36	NS
**Mean (SD)**	**17.25 (4.72)**				**434.5 (68.03)**	**23.95 (10.29)**	**8.45 (0.99)**	**21.15 (2.03)**	**0.48 (0.04)**	**0.85 (0.57)**	**36.25 (20.01)**	-

Abbreviations: GAD, glutamic acid decarboxylase; NS, not suspended; ND, non-determined.
a Since mobilization regimen;
b Patients 1, 21 and 25 presented diabetic ketoacidosis at diagnosis;
c Patients 1 and 20 used glucocorticoids in the conditioning regimen;
d Patients self-identified as having both black and white racial parentage;
e Patients lost follow-up.
Patients were numbered according to transplantation day.

Supplementary Table 4. Insulin-use status of T1D patients following non-myeloablative AHSCT.

Patient No./Sex	Age, y	Follow-up, mo[a]	Time free from insulin, mo	Insulin-use status						Groups[g]
				24 mo post-AHSCT	30 mo post-AHSCT	36 mo post-AHSCT	40 mo post-AHSCT	43 mo post-AHSCT	48 mo post-AHSCT	
1/M[b,c]	24	12[e]	NS	NS	NS	NS	NS	NS	NS	-
2/M	27	106	47 (T)	IF	IF	IF	IF	IF	**ID**	≥ 3.5 years
3/M	21	104	100 (C)	IF	IF	IF	IF	IF	IF	≥ 3.5 years
4/M	15	104	43 (T)	IF	IF	IF	IF	IF	**ID**	≥ 3.5 years
5/M	16	95	94 (C)	IF	IF	IF	IF	IF	IF	≥ 3.5 years
6/M	14	71[e]	60 (T)	IF	IF	IF	IF	IF	IF	≥ 3.5 years
7 /F	20	91	7 (T)	ID	ID	ID	ID	ID	ID	< 3.5 years
8/M	16	88	44 (T)	IF	IF	IF	IF	IF	**ID**	≥ 3.5 years
9/F	18	88	61 (T)	IF	IF	IF	IF	IF	IF	≥ 3.5 years
10/F	17	87	9 (T)	ID	ID	ID	ID	ID	ID	< 3.5 years
11/M	16	15[e]	12 (T)	ID	ID	ID	ID	ID	ID	< 3.5 years
12/F	14	80	66 (T)	IF	IF	IF	IF	IF	IF	≥ 3.5 years
13/M	24	79	67(T)	IF	IF	IF	IF	IF	IF	≥ 3.5 years
14/M	31	78	66(T)	IF	IF	IF	IF	IF	IF	≥ 3.5 years
15/M	16	77	9 (T)	ID	ID	ID	ID	ID	ID	< 3.5 years
16/M	16	66	29 (T)	IF	ID	ID	ID	ID	ID	< 3.5 years
17/M	17	65	64 (C)	IF	IF	IF	IF	IF	IF	≥ 3.5 years
18/M	21	38[e]	23 (T)	ID	ID	ID	ID	ID	ID	< 3.5 years
19/M	15	63	15(T)	ID	ID	ID	ID	ID	ID	< 3.5 years
20/M[c]	13	61	NS	NS	NS	NS	NS	NS	NS	-
21/F[b]	16	36[e]	NS	NS	NS	NS	NS	NS	NS	-
22/F	15	57	9 (T)	ID	ID	ID	ID	ID	ID	< 3.5 years
23/M	22	55	6 (T)	ID	ID	ID	ID	ID	ID	< 3.5 years
24/M	19	45	7 (T)	ID	ID	ID	ID	ID	ID	< 3.5 years
25/F[b]	16	36	NS	NS	NS	NS	NS	NS	NS	-

Abbreviations: AUC, area under the curve; C, continuously; NS, not suspended; T, transiently; IF, Insulin-Free; ID, Insulin-Dependent; AHSCT, autologous hematopoietic stem cell transplantation.

Supplementary Table 5. C-peptide levels in T1D patients submitted to AHSCT.

Period	Patients	N	Mean	SD	IC 95%		*P* Value[a]	*P* Value[b]	*P* Value[c]
					LI	LS			
**PreTx**	All	21	201.96	169.61	124.76	279.17	-	-	-
	<3.5years	10	277.25	191.05	140.58	413.92	-	-	-
	≥3.5years	11	133.52	117.73	54.43	212.61	-	-	.13
**6 mo**	All	20	400.54	276.80	270.99	530.08	**.003**	-	-
	<3.5years	10	382.75	235.96	213.95	551.54	-	.22	-
	≥3.5years	10	418.32	324.61	186.11	650.54	-	**.001**	.73
**12 mo**	All	21	413.76	197.67	323.78	503.74	**.001**	-	-
	<3.5years	10	365.76	170.42	243.85	487.66	-	.30	-
	≥3.5years	11	457.40	218.19	310.82	603.98	-	**<.001**	.34
**24 mo**	All	19	530.95	372.46	351.43	710.47	**<.001**	-	-
	<3.5years	8	309.01	157.22	177.57	440.45	-	.82	-
	≥3.5years	11	692.36	405.39	420.02	964.71	-	**<.001**	**<.001**
**36 mo**	All	17	459.78	246.51	333.04	586.53	**<.001**	-	-
	<3.5years	8	266.01	177.41	117.69	414.33	-	.72	-
	≥3.5years	9	632.02	151.89	515.27	748.78	-	**<.001**	**<.001**
**48 mo**	All	19	359.29	245.61	240.91	477.67	**.03**	-	-
	<3.5years	8	224.66	142.44	105.58	343.74	-	.43	-
	≥3.5years	11	457.21	263.31	280.31	634.11	-	**<.001**	**.01**
**60 mo**	All	15	366.68	154.06	281.36	451.99	.07	-	-
	<3.5years	5	265.38	112.48	125.71	405.04	-	.66	-
	≥3.5years	10	417.33	150.82	309.44	525.22	-	**.002**	.14
**72 mo**	All	11	245.30	100.33	177.90	312.70	.97	-	-
	<3.5years	2	139.58	42.30	-240.46	519.61	-	.41	-
	≥3.5years	9	268.80	94.57	196.11	341.49	-	.17	.54

Time course of total area under the curve (AUC) of C-peptide levels (ng/mL per 2h) during mixed-meal tolerance test in 11 patients insulin-free ≥ 3.5-years and in 10 patients insulin-free < 3.5-years groups. To convert C-peptide to nmol/L, multiply by 0.331. PreTx, pre-transplantation period; mo, months; T1D, type 1 diabetes. [a], all patients together in each period compared to PreTx period; [b], each patient group at cited period compared to its respective PreTx period; [c], comparison between groups in each period. Statistical analyses were performed using a model of multiple regression of mixed effects.

Supplementary Table 6. Cumulative frequency of islet-specific autoreactive CD8$^+$ T-cells in T1D patients following AHSCT.

Period	Patients	N	Mean	SD	IC 95%		*P* Value[a]	*P* Value[b]
					LI	LS		
**PreTx**	<3.5years	5	0.037	0.012	0.022	0.051	-	-
	≥3.5years	6	0.023	0.010	0.013	0.034	-	.09
**6 mo**	<3.5years	5	0.038	0.014	0.020	0.056	.81	-
	≥3.5years	6	0.022	0.013	0.009	0.036	.89	**.04**
**12 mo**	<3.5years	4	0.039	0.006	0.030	0.049	.99	-
	≥3.5years	5	0.017	0.008	0.008	0.027	.62	**.04**
**24 mo**	<3.5years	5	0.038	0.017	0.018	0.059	.81	-
	≥3.5years	6	0.025	0.012	0.012	0.038	.72	.10
**>2 years**	<3.5years	2	0.039	0.008	-0.037	0.115	.95	-
	≥3.5years	4	0.034	0.016	0.008	0.060	.19	.70
**>3 years**	<3.5years	5	0.039	0.004	0.035	0.044	.72	-
	≥3.5years	6	0.028	0.018	0.010	0.047	.40	.17

Frequencies (%) of islet-specific autoreactive CD8$^+$ T-cells were determined for each HLA-A2 positive patient before and after AHSCT. **PreTx**, pre-transplantation period; **mo**, months; T1D, type 1 diabetes. [a], each patient group at cited period compared to its respective PreTx period; [b], comparison between groups in each period. Statistical analyses were performed using a model of multiple regression of mixed effects.

Supplementary Table 7. Baseline cumulative frequency of islet-specific autoreactive CD8$^+$ T-cells in T1D patients.

Period	Patients	N	Mean	SD	IC 95%		*P* Value[a]	*P* Value[b]	*P* Value[c]
					LI	LS			
**PreTx**	All	12	0.0296	0.0118	0.0221	0.0371	-	-	-
	Low	6	0.0200	0.0054	0.0143	0.0257	-	-	-
	High	6	0.0392	0.0077	0.0311	0.0472	-	-	**.001**
**6 mo**	All	12	0.0293	0.0147	0.0199	0.0386	.93	-	-
	Low	6	0.0175	0.0045	0.0128	0.0222	-	.66	-
	High	6	0.0410	0.0112	0.0292	0.0528	-	.74	**<.001**
**12 mo**	All	10	0.0265	0.0127	0.0174	0.0356	.54	-	-
	Low	5	0.0174	0.0079	0.0076	0.0272	-	.66	-
	High	5	0.0356	0.0097	0.0235	0.0477	-	.55	**.005**
**24 mo**	All	11	0.0313	0.0153	0.0210	0.0415	.71	-	-
	Low	6	0.0200	0.0068	0.0129	0.0271	-	.99	-
	High	5	0.0448	0.0103	0.0320	0.0576	-	.34	**<.001**
**>2 years**	All	6	0.0358	0.0135	0.0216	0.0500	.36	-	-
	Low	3	0.0290	0.0155	-0.0096	0.0676	-	.19	-
	High	3	0.0427	0.0087	0.0210	0.0644	-	.61	.09
**>3 years**	All	11	0.0333	0.0138	0.0240	0.0426	.39	-	-
	Low	6	0.0282	0.0174	0.0099	0.0464	-	.15	-
	High	5	0.0394	0.0036	0.0350	0.0438	-	.97	.06

Frequencies (%) of islet-specific autoreactive CD8$^+$ T-cells were determined for each HLA-A2 positive patient before the AHSCT procedure (baseline). Patients were divided in two groups, High or Low cytotoxic T-lymphocyte (CTL) autoreactivity at baseline. **PreTx**, pre-transplantation period; **mo**, months; T1D, type 1 diabetes. [a], all patients together in each period compared to PreTx period; [b], each patient group at cited period compared to its respective PreTx period; [c], comparison between groups in each period. Statistical analyses were performed using a model of multiple regression of mixed effects.

Supplementary Table 8. C-peptide levels in T1D patients with High or Low CLT autoreactivity at baseline.

Period	Patients	N	Mean	SD	IC 95%		*P* Value[a]	*P* Value[b]	*P* Value[c]
					LI	LS			
**PreTx**	All	12	263.14	244.90	107.54	418.74	-	-	-
	Low	6	212.92	195.18	8.09	417.74	-	-	-
	High	6	313.37	296.30	2.42	624.32	-	-	.45
**6 mo**	All	10	432.15	293.44	222.23	642.06	.09	-	-
	Low	5	470.48	376.25	3.31	937.66	-	.06	-
	High	5	393.81	220.22	120.37	667.24	-	.56	.60
**12 mo**	All	12	398.79	197.36	273.39	524.19	.14	-	-
	Low	6	519.18	127.64	385.24	653.13	-	**.02**	-
	High	6	278.40	186.05	83.15	473.65	-	.79	.07
**24 mo**	All	12	485.20	279.89	307.36	663.03	**.02**	-	-
	Low	6	656.27	157.31	491.19	821.35	-	**.001**	-
	High	6	314.12	278.14	22.24	606.01	-	.99	**.01**
**36 mo**	All	10	440.44	291.16	232.16	648.72	.09	-	-
	Low	5	587.04	277.77	242.14	931.94	-	**.008**	-
	High	5	293.84	244.65	-9.94	597.61	-	.87	**.04**
**48 mo**	All	11	375.89	260.86	200.64	551.13	.26	-	-
	Low	6	476.06	298.25	163.06	789.05	-	**.04**	-
	High	5	255.68	160.66	56.19	455.17	-	.66	.11
**60 mo**	All	9	370.17	182.18	230.14	510.21	.39	-	-
	Low	6	441.21	186.06	245.96	636.47	-	.08	-
	High	3	228.10	28.23	157.97	298.22	-	.56	.18
**72 mo**	All	7	227.32	116.12	119.92	334.72	.60	-	-
	Low	4	296.64	106.68	126.89	466.39	-	.60	-
	High	3	134.89	30.99	57.91	211.88	-	.25	.35

Frequencies (%) of islet-specific autoreactive $CD8^+$ T-cells were determined for each HLA-A2 positive patient before the AHSCT procedure (baseline). Patients were divided in two groups, High or Low cytotoxic T-lymphocyte (CTL) autoreactivity at baseline. Table shows the time course of total area under the curve (AUC) of C-peptide levels (ng/mL per 2h) during mixed-meal tolerance test in 6 patients with High and in 6 patients with Low cytotoxic T-lymphocyte (CTL) autoreactivity at baseline. To convert C-peptide to nmol/L, multiply by 0.331. PreTx, pre-transplantation period; mo, months; T1D, type 1 diabetes. [a], all patients together in each period compared to PreTx period; [b], each patient group at cited period compared to its respective PreTx period; [c], comparison between groups in each period. Statistical analyses were performed using a model of multiple regression of mixed effects.

frontiers

Supplementary Table 9. T-cell receptor excision circles levels in peripheral blood mononuclear cells of T1D patients following AHSCT.

Period	Patients	N	Mean	SD	IC 95%		*P* Value[a]	*P* Value[b]	*P* Value[c]
					LI	LS			
**PreTx**	All	12	0.00340	.0190	0.00219	0.00461	-	-	-
	<3.5years	7	0.00342	0.00171	0.00185	0.00500	-	-	-
	≥3.5years	5	0.00337	0.00236	0.00044	0.00630	-	-	.63
**3 mo**	All	7	0.00038	0.00032	0.00008	0.00067	**<.001**	-	-
	<3.5years	3	0.00071	0.00005	0.00059	0.00083	-	**.002**	-
	≥3.5years	4	0.00013	0.00011	-0.00004	0.00029	-	**<.001**	**.04**
**6 mo**	All	8	0.00220	0.00290	-0.00022	0.00463	.06	-	-
	<3.5years	2	0.00158	0.00007	0.00098	0.00217	-	.24	-
	≥3.5years	6	0.00241	0.00340	-0.00116	0.00598	-	.10	.65
**12 mo**	All	10	0.00574	0.00470	0.00238	0.00911	.06	-	-
	<3.5years	3	0.00747	0.00661	-0.00895	0.02390	-	.31	-
	≥3.5years	7	0.00500	0.00406	0.00125	0.00875	-	.23	.68
**18 mo**	All	10	0.01136	0.01386	0.00145	0.02128	**.001**	-	-
	<3.5years	4	0.01666	0.02206	-0.01844	0.05177	-	**.03**	-
	≥3.5years	6	0.00783	0.00407	0.00356	0.01210	-	**.03**	.63
**24 mo**	All	11	0.01207	0.01132	0.00447	0.01967	**<.001**	-	-
	<3.5years	4	0.01422	0.01177	-0.00451	0.03296	-	**.007**	-
	≥3.5years	7	0.01084	0.01180	-0.00008	0.02176	-	**.04**	.28
**30 mo**	All	9	0.00944	0.00420	0.00621	0.01267	**.001**	-	-
	<3.5years	3	0.00927	0.00626	-0.00629	0.02484	-	.19	-
	≥3.5years	6	0.00953	0.00354	0.00581	0.01324	-	**.01**	.71
**36 mo**	All	10	0.01310	0.01306	0.00375	0.02244	**<.001**	-	-
	<3.5years	3	0.02638	0.01824	-0.01894	0.07170	-	**<.001**	-
	≥3.5years	7	0.00740	0.00434	0.00338	0.01142	-	**.02**	.05
**42 mo**	All	4	0.02795	0.04011	-0.03588	0.09178	**.002**	-	-
	<3.5years	2	0.04695	0.05776	-0.47202	0.56591	-	**.007**	-
	≥3.5years	2	0.00895	0.00686	-0.05267	0.07057	-	.12	.29
**48 mo**	All	6	0.01251	0.00903	0.00304	0.02199	**<.001**	-	-
	<3.5years	3	0.01225	0.00330	0.00406	0.02043	-	**.01**	-
	≥3.5years	3	0.01278	0.01388	-0.02171	0.04726	-	**.004**	.88
**54 mo**	All	6	0.01543	0.02060	-0.00619	0.03705	**<.001**	-	-
	<3.5years	2	0.03011	0.03770	-0.30864	0.36886	-	**.03**	-
	≥3.5years	4	0.00809	0.00425	0.00133	0.01486	-	**.009**	.70
**60 mo**	All	10	0.01545	0.01220	0.00672	0.02417	**<.001**	-	-
	<3.5years	3	0.02151	0.01189	-0.00803	0.05105	-	**<.001**	-
	≥3.5years	7	0.01285	0.01224	0.00153	0.02417	-	**<.001**	.11

T cell receptor excision circles (TREC) levels are expressed as TREC molecules/100 ng DNA of peripheral blood mononuclear cells. PreTx, pre-transplantation period; mo, months; T1D, type 1 diabetes. [a], all patients together in each period compared to PreTx period; [b], each patient group at cited period compared to its respective PreTx period; [c], comparison between groups in each period. Statistical analyses were performed using a model of multiple regression of mixed effects.

frontiers

Supplementary Table 10. T cell repertoire diversity in T1D patients following AHSCT.

Period	Patients	N	Mean	SD	IC 95%		*P* Value[a]	*P* Value[b]	*P* Value[c]
					LI	LS			
**PreTx**	All	18	158.78	24.05	146.82	170.74	-	-	-
	<3.5years	9	153.89	25.15	134.56	173.22	-	-	-
	≥3.5years	9	163.67	23.30	145.76	181.57	-	-	.44
**6 mo**	All	17	134.41	40.56	113.56	155.27	**.006**	-	-
	<3.5years	7	124.43	43.22	84.46	164.40	-	**.04**	-
	≥3.5years	10	141.40	39.34	113.26	169.54	-	.07	.23
**12 mo**	All	18	148.78	26.64	135.53	162.03	.22	-	-
	<3.5years	7	136.00	23.60	114.17	157.83	-	.17	-
	≥3.5years	11	156.91	26.19	139.32	174.50	-	.55	.12
**18 mo**	All	19	139.89	27.51	126.63	153.15	**.03**	-	-
	<3.5years	8	137.00	33.09	109.34	164.66	-	.19	-
	≥3.5years	11	142.00	24.17	125.76	158.24	-	.07	.69
**24 mo**	All	17	149.65	22.67	137.99	161.30	.29	-	-
	<3.5years	8	144.63	26.07	122.83	166.42	-	.46	-
	≥3.5years	9	154.11	19.63	139.02	169.20	-	.45	.46
**30 mo**	All	15	127.73	30.74	110.71	144.76	**.001**	-	-
	<3.5years	8	124.13	27.51	101.12	147.13	-	**.02**	-
	≥3.5years	7	131.86	35.84	98.71	165.01	-	**.02**	.62
**36 mo**	All	11	126.55	29.70	106.60	146.50	**.001**	-	-
	<3.5years	4	132.75	35.66	76.01	189.49	-	.21	-
	≥3.5years	7	123.00	28.17	96.95	149.05	-	**.003**	.53
**42 mo**	All	11	121.55	21.13	107.35	135.74	**<.001**	-	-
	<3.5years	4	113.00	12.19	93.60	132.40	-	**.01**	-
	≥3.5years	7	126.43	24.36	103.90	148.96	-	**.007**	.40
**48 mo**	All	12	119.00	26.26	102.31	135.69	**<.001**	-	-
	<3.5years	5	123.80	36.66	78.28	169.32	-	**.03**	-
	≥3.5years	7	115.57	18.31	98.64	1331	-	**<.001**	.65
**54 mo**	All	6	113.00	37.48	73.67	152.33	**<.001**	-	-
	<3.5years	2	88.00	22.63	-115.30	291.30	-	**.002**	-
	≥3.5years	4	125.50	39.31	62.95	188.05	-	**.02**	.10
**60 mo**	All	13	103.77	17.85	92.98	114.56	**<.001**	-	-
	<3.5years	3	104.67	4.51	93.47	115.87	-	**.006**	-
	≥3.5years	10	103.50	20.50	88.84	118.16	-	**<.001**	.96

T cell repertoire diversity is calculted by the Complexity Score (CS) as explained in the On-line Methods. PreTx, pre-transplantation period; mo, months; T1D, type 1 diabetes. [a], all patients together in each period compared to PreTx period; [b], each patient group at cited period compared to its respective PreTx period; [c], comparison between groups in each period. Statistical analyses were performed using a model of multiple regression of mixed effects.
